# RNF13 Dileucine Motif Variants L311S and L312P Interfere with Endosomal Localization and AP-3 Complex Association

**DOI:** 10.3390/cells10113063

**Published:** 2021-11-06

**Authors:** Valérie C. Cabana, Antoine Y. Bouchard, Audrey M. Sénécal, Kim Ghilarducci, Saïd Kourrich, Laurent Cappadocia, Marc P. Lussier

**Affiliations:** 1 Département de Chimie, Université du Québec à Montréal, Montréal, QC H2X 2J6, Canada; cabana.valerie@courrier.uqam.ca (V.C.C.); bouchard.antoine.2@courrier.uqam.ca (A.Y.B.); senecal.audrey.3@courrier.uqam.ca (A.M.S.); ghilarducci.kim@courrier.uqam.ca (K.G.); cappadocia.laurent@uqam.ca (L.C.); 2 Centre d’Excellence en Recherche sur les Maladies Orphelines—Fondation Courtois (CERMO-FC), Université du Québec à Montréal, Montréal, QC H2X 3Y7, Canada; kourrich.said@uqam.ca; 3 Département des Sciences Biologiques, Université du Québec à Montréal, Montréal, QC H2X 1Y4, Canada; 4 Department of Psychiatry, University of Texas Southwestern Medical Center, Dallas, TX 75390, USA

**Keywords:** RNF13, AP-3 complex, endosomes, lysosomes, intracellular trafficking, genetic mutations, developmental and epileptic encephalopathy-73

## Abstract

Developmental and epileptic encephalopathies (DEE) are rare and serious neurological disorders characterized by severe epilepsy with refractory seizures and a significant developmental delay. Recently, DEE73 was linked to genetic alterations of the RNF13 gene, which convert positions 311 or 312 in the RNF13 protein from leucine to serine or proline, respectively (L311S and L312P). Using a fluorescence microscopy approach to investigate the molecular and cellular mechanisms affected by RNF13 protein variants, the current study shows that wild-type RNF13 localizes extensively with endosomes and lysosomes, while L311S and L312P do not extensively colocalize with the lysosomal marker Lamp1. Our results show that RNF13 L311S and L312P proteins affect the size of endosomal vesicles along with the temporal and spatial progression of fluorescently labeled epidermal growth factor, but not transferrin, in the endolysosomal system. Furthermore, GST-pulldown and co-immunoprecipitation show that RNF13 variants disrupt association with AP-3 complex. Knockdown of AP-3 complex subunit AP3D1 alters the lysosomal localization of wild-type RNF13 and similarly affects the size of endosomal vesicles. Importantly, our study provides a first step toward understanding the cellular and molecular mechanism altered by DEE73-associated genetic variations of RNF13.

## 1. Introduction

The human genome encodes approximately 600 ubiquitin (Ub) E3 ligases [1,2]. These Ub ligases are crucial for bringing specificity to the protein ubiquitination process by recognizing the appropriate substrate protein [2,3,4]. Specifically, protein ubiquitination is a post-translational modification that is implicated in various cellular processes, such as protein trafficking, cell-cycle regulation, DNA repair, apoptosis, and signal transduction [5,6,7]. Ubiquitination is necessary for protein degradation, and the ubiquitin–proteasome system (UPS) is critical to maintain protein homeostasis by selectively degrading abnormally folded or damaged proteins that could become toxic [8]. Dysfunction of this pathway is implicated in the pathogenesis of multiple diseases, such as Angelman syndrome [9,10], Alzheimer’s disease [11,12], Parkinson’s disease [13,14], and in many types of cancer [15,16,17].

E3 ligases can be classified into three families based on their catalytic core domain: the Homology to E6AP Carboxyl Terminus (HECT), the Really Interesting New Gene (RING) finger, and the U-box protein families [18]. Among the several hundred Ub E3 ligases characterized by the presence of a RING domain [19], 49 of those contain integral transmembrane domains [20,21,22]. A phylogenetic tree constructed after analysis of the human transmembrane RING-domain Ub ligases presents subdivisions that include members of the tripartite motif-containing (TRIM), the protease-associated (PA) transmembrane (TM) RING (PA-TM-RING), the RING between RING (RBR), and the membrane-associated RING-CH (MARCH) families [21]. Specifically, the PA-TM-RING family is characterized by the presence of a protease-associated domain, a single transmembrane domain, a RING-H2 domain, and usually a putative N-terminal signal peptide [21,23]. While the RING domain is needed for the ligase activity of the protein, the PA domain is a conserved sequence proposed to mediate protein–protein interaction [21,23]. Importantly, many members of the PA-TM-RING family, such as RNF167 [24,25,26] or RNF13 [23,27], localize to endosomal membranes where they can potentially impact important and complex biological events.

The RING finger protein 13 (*RNF13*) gene is evolutionarily conserved in many invertebrate and vertebrate organisms, including humans. Over the years, the RNF13 protein has been shown to localize in the endoplasmic reticulum (ER), late endosomes and lysosomes, and at the plasma membrane [23,28,29,30]. RNF13 was first identified as chicken RING zinc finger (C-RZF) and its expression was found to be upregulated when chicken embryo brain cells were treated with the extracellular matrix component tenascin-C [31]. RNF13 plays a role in myogenesis, tumorigenesis, and cell proliferation [29,32,33]. Recent studies have shown that *RNF13* expression in atherosclerosis plaques is upregulated [34], while it is downregulated in uveal melanoma [35] and asthma [36]. Ectopic expression of RNF13 inhibits myoblast proliferation, whereas treatment with myostatin, a muscle growth inhibitor, upregulates *RNF13* expression [32]. In PC12 cells, ectopic expression of RNF13 promoted the spontaneous growth of neurites [37]. Treatment of B35 neuroblastoma cells with dibutyryl-cAMP, an agent that stimulates neurite extension, increased endogenous RNF13 mRNA expression two-fold [28]. Furthermore, RNF13 Ub ligase activity promotes the ubiquitination of SNARE-associated protein (snapin), allowing its interaction with synaptosome-associated protein 25 (SNAP-25) to strengthen the binding between synaptotagmin I and SNAP-25. This interaction is crucial for the assembly of the soluble N-ethylmaleimide-sensitive factor attachment protein receptor (SNARE) complex and the release of neurotransmitters into the synaptic cleft [38].

Recent advances in genome sequencing and the access to public databases eased the identification of various mutations within the genes of patients. For instance, consultation of the BioMuta dataset [39], which catalogues single-nucleotide variations in cancer, currently displays more than 60 unique variations within the coding sequence of the human *RNF13* gene. RNF13 mutations were identified across different cancer types. Some RNF13 variant proteins resulting from somatic mutations were characterized for their involvement in tumorigenesis [23]. Additionally, a study reported that three unrelated individuals with an infantile neurodegenerative disorder were carrying de novo heterozygous mutations within the *RNF13* gene (c.932T > C (p.L311S) and c.935T > C (p.L312P)) [40]. Clinical features include, but are not limited to, abnormally increased muscle tone, refractory epilepsy, microcephaly, and profound intellectual disability. In this specific study, fibroblasts and lymphoblasts derived from an affected individual carrying the RNF13 L311S variant protein were more prone to ER stress [40]. In this context, further characterization of RNF13 variants L311S and L312P would lead to a better understanding of altered cellular mechanisms. The present study demonstrates that RNF13 L311S and L312P variant proteins do not localize in the ER. Instead, while wild-type (WT) RNF13 protein predominantly localizes in late endosomes and lysosomes, the presence of RNF13 L311S and L312P variants in lysosomal vesicles is diminished. Cells expressing RNF13 variants display enlarged endolysosomal vesicles when compared to RNF13 WT. Moreover, this study provides evidence that RNF13 L311S and L312P alter epidermal growth factor (EGF) sorting. Finally, L311S and L312P variants disrupt RNF13 binding to subunits of the AP-3 complex while AP-3 knockdown alters the lysosomal targeting of RNF13. Overall, this work provides the first glimpse into an altered endolysosomal pathway due to the expression of RNF13 L311S and L312P variant proteins.

## 2. Materials and Methods

### 2.1. Antibodies

The following antibodies were used: Rabbit anti-HA (IF and WB: 1:1000, New England Biolabs Ltd., Whitby, ON, Canada, Cat# 3724, RRID:AB_1549585), Mouse anti-HA (IF: 1:1000, BioLegend, San Diego, CA, USA, Cat# 901502, RRID:AB_2565007), Mouse anti-GFP (IF: 1:1000, Invitrogen, Carlsbad, CA, USA, Cat #MA5-15256, RRID:AB_10979281), Rabbit anti-AIF (IF: 1:1000, New England Biolabs Ltd., Cat# 5318, RRID:AB_10634755), Rabbit anti-EEA1 (IF: 1:500, New England Biolabs Ltd., Cat# 3288, RRID:AB_2096811), Rabbit anti-Lamp1 (IF: 1:500, New England Biolabs Ltd., Cat# 9091, RRID:AB_2687579), Rabbit anti-PDI (IF: 1:250, New England Biolabs Ltd., Cat# 3501, RRID:AB_215643), Rabbit anti-RCAS1 (IF: 1:250, New England Biolabs Ltd., Cat# 12290, RRID:AB_2736985), Mouse anti-Rab5A (IF: 1:800, New England Biolabs Ltd., Cat# 46449, RRID:AB_2799303), Rabbit anti-Rab7 (IF: 1:200, New England Biolabs Ltd., Cat# 9367, RRID:AB_1904103), Rabbit anti-Rab9 (IF: 1:200, New England Biolabs Ltd., Cat# 5118, RRID:AB_10621426), Rabbit anti-AP3S1 (WB: 1:500, Bio-Techne Canada, Toronto, ON, Canada, Cat# NBP1-76588, RRID:AB_11018734), Rabbit anti-AP3D1 (WB: 1:1000, Bio-Techne Canada, Cat# NBP2-92046), AlexaFluor 405-conjugated Goat anti-Mouse (1:1000, Thermo Fisher Scientific, Saint-Laurent, QC, Canada, Cat# A-31553, RRID:AB_221604), AlexaFluor 488-conjugated Goat anti-Mouse (1:1000, Thermo Fisher Scientific, Cat# A-11017, RRID:AB_253408), AlexaFluor 647-conjugated Goat anti-Mouse (1:1000, Thermo Fisher Scientific, Cat# A31626, RRID:AB_2535792), AlexaFluor 488-conjugated Goat anti-Rabbit (1:1000, Thermo Fisher Scientific, Cat# A-11070, RRID:AB_2534114), AlexaFluor 647-conjugated Goat anti-Rabbit (1:1000, Thermo Fisher Scientific, Cat# A31634), and horseradish peroxidase (HRP)-conjugated Goat anti-Rabbit (1:10000, New England Biolabs Ltd., Cat# 7074, RRID:AB_2099233).

### 2.2. Molecular Biology

GenScript (Piscataway, NJ, USA) synthesized wild-type, full-length human RNF13 transcript variant 1 (accession NM_007282.4) that included the natural Kozak sequence and a C-terminus HA tag. This construct was cloned into pUC57 vector and served as the template for generating protein variants L311S, L312P, and L311L312/AA via site-directed mutagenesis. WT and mutated cDNAs were excised using standard enzymatic restriction strategies and ligated into BamHI and XbaI sites of pcDNA3.0 (+) (Thermo Fisher Scientific) for immunofluorescence assay and into NheI and XhoI sites of pCAGGS-IRES-mCherry for immunoprecipitation assay. The pUC57 vector was used as template for generating C-terminal RNF13 WT and variant proteins (a.a. 281–381). PCR was performed with Q5 high-fidelity DNA polymerase (New England BioLabs Ltd., Cat# M0492S). Fragments were excised using standard enzymatic restriction strategies and ligated into EcoRI and XhoI sites of pGEX-4T-1 (GE Healthcare Life Sciences, now known as Cytiva, Mississauga, ON, Canada). All plasmids were confirmed by Sanger sequencing (Genome Quebec, Montreal, QC, Canada).

### 2.3. Cell Culture and Transfection

HeLa (from Diana Alison Averill, Montreal, QC, Canada) and HEK293 T/17 cells from the American Type Culture Collection (ATCC, Gaithersburg, MD, USA, Cat# CRL-11268) were cultured at subconfluence in Dulbecco’s modified Eagle medium (DMEM, Thermo Fisher Scientific, Cat# 11995-065) containing 10% fetal bovine serum (FBS, VWR Life Science, Mississauga, ON, Canada, Cat# MPCA97068-085) in 5% CO_2_-containing humidified air at 37 °C. Cells were not recently authenticated nor tested for mycoplasma contamination. For immunostaining and trafficking assay, 24 h prior to transfection, 6 × 10^4^ HeLa cells were seeded in a 24-well plate containing a 12 mm round glass coverslip #1.5 (UltiDent Scientific, Saint-Laurent, QC, Canada, Cat# 170-C12MM). Two hours before transfection, the culture medium was replaced with fresh medium. For transfection, a mixture containing 0.4 µg of total plasmid DNA and 250 mM CaCl_2_ was added dropwise to an equal volume of 2× Hanks’ Balanced Salt Solution to form a calcium phosphate–DNA precipitate. After a 20 min incubation at room temperature, the calcium–DNA complexes were added to the cells, which were further incubated for 24 h at 37 °C with 5% CO_2_. When required, ER stress was induced in transfected HeLa cells using 1.25 µg/mL tunicamycin (Cayman Chemical, Ann Arbor, MI, USA, Cat# 11445), 1 µM thapsigargin (Cayman Chemical, Cat# 10522), or incubated with an equal volume of dimethyl sulfoxide for 3 h before performing immunostaining as described below. For immunoprecipitation assay, HEK293 T/17 cells were transfected using Lipofectamine 2000 in poly-D-lysine-treated six-well plates as described previously [41].

### 2.4. Immunostaining

Transfected HeLa cells were washed with phosphate-buffered saline (PBS) and fixed using ice-cold 4% paraformaldehyde (PFA)/4% sucrose in PBS for 15 min at room temperature. Cells were permeabilized with 0.25% Triton X-100 in PBS for 15 min before blocking non-specific sites with 10% normal goat serum (NGS) in PBS for 1 h. Coverslips were incubated with primary antibodies diluted in 3% NGS/PBS for 1 h, followed by incubation with the appropriate conjugated secondary antibodies also diluted in 3% NGS/PBS for 1 h. Coverslips were washed in PBS, mounted using ProLong Diamond Antifade (Thermo Fisher Scientific, Cat# P36961) and kept at 4 °C before performing image acquisition.

### 2.5. Uptake and Trafficking of EGF and Transferrin

Transfected HeLa cells were starved in DMEM supplemented with 0.2% bovine serum albumin (BSA) for 2 h at 37 °C. A total of 0.5 µg/mL AlexaFluor 647 EGF complex (Thermo Fisher Scientific, Cat# E35351) or 10 µg/mL CF640R-conjugated Human Transferrin (Biotium, Fremont, CA, USA, Cat# 00085) were added to the cells and incubated at 37 °C for 5 min (pulse). For the chase, cells were incubated in fresh culture medium (DMEM + 10% FBS) at 37 °C for a period up to 60 min. Cells were washed in PBS and fixed in PFA immediately after the chase period. Once all the coverslips were fixed, the immunostaining method was performed as described above.

### 2.6. Fluorescence Microscopy Image Acquisition

Image acquisition was performed by using an inverted epi-fluorescence microscope Olympus IX83 equipped with a U Plan S-Apo 60×/1.35 numerical aperture oil objective (Olympus Canada Inc., Richmond Hill, ON, Canada), the X-Cite Xylis 365 LED-based illumination source (Excelitas Technologies Corp., Waltham, MA, USA), and the Zyla 4.2 Plus sCMOS camera (Andor, Concord, MA, USA). The Olympus CellSens Dimension software controlled the system during image acquisition. Z-stack images were acquired with 7 to 12 optical slices taken at 0.27 µm intervals with resolution set at 2048 × 2048 pixels. Intensity and exposure time for illuminating the samples were set to obtain the highest signal possible without reaching saturation. Epifluorescence images were deconvolved using the Olympus 3D Deconvolution feature in the Olympus CellSens Dimension software.

### 2.7. Image Analysis

To analyze vesicle size, the diameter of the five largest vesicles per cell was measured in a deconvoluted single optical slice using the Measure—Arbitrary Line function in Olympus CellSens Dimension software. To analyze colocalization between RNF13 and endosomal markers, a deconvoluted single optical slice was separated in two channels. The threshold was adjusted to have signals only from puncta, and the Manders overlap coefficient was obtained using the JaCoP plugin in Fiji. To analyze the uptake and trafficking of EGF and transferrin, all optical sections were Z-stacked using the maximum intensity option in ImageJ, and the threshold was adjusted to have signals only from puncta. Afterward, puncta were counted using the Fiji’s Analyze Particle function. Pearson’s colocalization coefficient between early endosome marker EEA1 and either EGF or transferrin was calculated using the JaCoP plugin in Fiji. For this analysis, all deconvoluted optical slices were Z-stacked using the maximum intensity option.

### 2.8. Statistical Analysis

At least three independent, non-blinded experiments were performed for all assays. After performing image analysis, datasets collected in Microsoft Excel were subjected to statistical analyses using GraphPad Prism 8. Outliers were excluded from datasets when identified using the ROUT method set to 1%. Because our sample size was relatively small, nonparametric tests were selected. Statistical difference was determined using either one-way ANOVA with the Kruskal–Wallis test or using two-way ANOVA with multiple comparisons.

### 2.9. TOPP2 E. coli Transformation and Culture

Bacteria were transformed using 50 ng of pGEX-4T-1 vector containing the coding sequence for RNF13 residues 281–381 and 20 µL of chemocompetent TOPP2 E. coli cells (Novagen). Transformants were selected on solid LB plates (10 g/L tryptone, 5 g/L yeast extract, 10 g/L NaCl, 15g/L agar) containing 100 µg/mL of ampicillin. One colony was randomly selected to inoculate 100 mL of liquid LB medium containing 100 µg/mL of ampicillin. This culture was incubated for 18 h at 28 °C and subsequently divided into 3 flasks, each containing 1L of SB medium (35 g/L tryptone, 20 g/L yeast extract, 5 g/L NaCl, pH 7.35) with 100 µg/mL ampicillin. This culture was incubated at 37 °C until it reached an OD600 of 1. Induction was performed with 0.3 mM isopropyl-β-d-thiogalactoside (IPTG) and cells were incubated for an additional 2 h at 30 °C. Cells were pelleted by centrifugation at 11,899 g at 4 °C for at least 40 min and re-suspended in a solution containing 20% sucrose, 50 mM Tris–HCl pH 8.0, and 100 µg/mL lysozyme using a ratio of 2 mL of solution per gram of bacterial cells. This solution was snap-frozen in liquid nitrogen and kept at −20 °C until use.

### 2.10. Purification of Recombinant C-Terminal RNF13 from TOPP2

The solution containing the bacterial cells was thawed at room temperature and its composition was adjusted to include 150 mM NaCl, 1 mM beta-mercaptoethanol, 1 mM phenylmethylsulfonyl fluoride (PMSF), and 5 mM ethylenediaminetetraacetic acid (EDTA). Sonication was performed for 3 cycles of 2 min with a minimum of 2 min in between cycles using a Branson S-450A sonifier equipped with a 0.5” horn. Power was adjusted to 50% with a 50% duty cycle. The resulting cell lysate was centrifuged at 48,384× *g* for 20 min at 4 °C. Supernatant was filtered using a 0.22 µm filter and the C-terminal portion of RNF13 was purified using a 1 mL GSTrap 4b column (Cytiva) and an AKTA start Fast Protein Liquid Chromatography (FPLC; Cytiva). The column was equilibrated using 10 column volumes (CV) of washing buffer composed of 20 mM Tris–HCl pH 8.0, 150 mM NaCl, 5 mM EDTA, 1 mM PMSF, and 1 mM BME. The cell lysate was then loaded on the column at a 0.5 mL/min flow rate. The column was washed using 10 CV of washing buffer at a 1.0 mL/min flow rate and elution was performed at a 0.5 mL/min using an Elution Buffer composed of 50 mM Tris–HCl pH 8.0, 20 mM reduced glutathione, and 1 mM PMSF. Elution fractions were combined and loaded on HiLoad 16/600 Superdex 75 (Cytiva) equilibrated with a gel filtration buffer composed of 20 mM Tris–HCl pH8.0, 150 mM NaCl, and 5 mM BME. Separation was performed using an AKTA Pure 25M FPLC (Cytiva). Fractions that contain RNF13 were pooled, snap-frozen in liquid nitrogen, and kept at −80 °C until needed.

### 2.11. GST Pulldown and Immunoprecipitation Assays

For GST pulldown, 10 cm plates with HEK293T/17 cells were washed twice with PBS and lysed in 3 mL ice-cold lysis buffer composed of 20 mM Tris–HCl pH 7.5, 150 mM NaCl, 1% Triton X-100, and 1X protease inhibitor EDTA-free cocktail (Cedarlane, Burlington, ON, Canada, Cat# B14001(CA)). Cells were collected and agitated at 15 rpm for 20 min at 4 °C. The sample was then centrifuged at 21,000 g for 15 min. A total of 250 uL of lysate was incubated overnight at 4 °C with 15 uL of glutathione–sepharose 4B resin (GE Healthcare, Chicago, IL, USA) pre-equilibrated in ice-cold lysis buffer and 25 ug of purified GST or GST-RNF13 proteins. After washing with ice-cold lysis buffer, proteins were eluted with Laemmli buffer before loading on SDS–PAGE. For immunoprecipitation assay, 6-well plates containing transfected HEK293T/13 cells were lysed with 1 mL ice-cold lysis buffer supplemented with 0.5% sodium deoxycholate (IP buffer). The lysis method was performed as described above. A total of 600 uL of lysate was incubated for 2 h at 4 °C with anti-HA affinity gel (Cedarlane, Cat# B23302(CA)) equilibrated in ice-cold IP buffer. Washes and elution were performed as described above.

### 2.12. SDS–PAGE and Western Blot

Proteins samples were separated on SDS–PAGE containing 0.5% of 2,2,2-trichloroethanol (TCE). The proteins were then transferred to a 0.45 um PVDF membrane using Trans-Blot Turbo system (Bio-Rad, Mississauga, ON, Canada) for 10 min, at 25V and 2.5A. Proteins on both gels and membranes were visualized using the *stainfree* mode of the ChemiDoc MP imaging System (Bio-Rad) and Bio-Rad Image Lab software. Subsequently, membranes were blocked for 1h at room temperature in 5% skim milk dissolved in TBS-T (20 mM Tris–HCl pH 7.5, 140 mM NaCl, 0.3% Tween-20). Incubation with primary antibody diluted in TBS-T containing 0.05% NaN_3_ was performed overnight at 4 °C, while incubation with horseradish peroxidase-coupled secondary antibody diluted in TBS-T was performed for 1 h at room temperature. A total of 3×3 min washes were performed between each step. Finally, immune complexes were revealed with Clarity Western ECL substrate (BioRad, Cat# 1705060) and chemiluminescent signal was obtained using the ChemiDoc MP imaging system.

### 2.13. Predicted Structure

The structure of the complex between RNF13 (Uniprot accession O43567) and AP3S1 (Uniprot accession Q92572) was modeled using the standalone version of AlphaFold [42]. Briefly, RNF13 and AP3S1 were combined into a single polypeptide chain separated by 15 glycine residues to ensure independent folding. Visualisation of the RNF13–AP3S1 complex was performed using the PyMOL Molecular Graphics System, Version 2.0 Schrödinger, LLC.

### 2.14. AP3D1 Knockdown

TriFECTa Kit Dicer-substrate siRNAs (dsiRNA) duplexes against Homo sapiens adaptor-related protein complex 3 subunit delta 1 (AP3D1, NM_003938) were purchased from Integrated DNA Technology (IDT, Coralville, IA). Specifically, we acquired the negative control scrambled dsiRNA (Cat# DS NC1) or AP3D1-specific duplex sequences #1 (5′-rCrGrCrUrArCrUrGrArGrCrArArCrUrUrGrUrUrArGrArAGA-3′ and 5′- rUrCrUrUrCrUrArArCrArArGrUrUrGrCrUrCrArGrUrArGrCrGrUrG-3′), #2 (5′-rGrUrCrArUrUrUrGrUrUrGrCrGrUrUrGrArArUrUrArUrCTG-3′ and 5′- rCrArGrArUrArArUrUrCrArArCrGrCrArArCrArArArUrGrArCrCrU-3′), and #3 (5′-rUrUrArCrArGrArUrGrUrUrGrGrGrArUrArCrGrArCrArUCA-3′ and 5′- rUrGrArUrGrUrCrGrUrArUrCrCrCrArArCrArUrCrUrGrUrArArArU-3′). Briefly, 4 h prior to transfection, 1.25 × 10^5^ HeLa cells were seeded in a 24-well plate containing a 12 mm round glass coverslip #1.5 (UltiDent Scientific, Cat# 170-C12MM) (for IF) or 2.5 × 10^5^ HeLa cells were seeded in a 6-well plate (for WB). A total of 10 nM DsiRNA was diluted in 50 uL Opti-MEM I and added to 0.5 uL (24-well) or 2 uL (6-well) of Lipofectamine 2000 transfection reagent diluted in 50 uL Opti-MEM I. The mixture was incubated for 20 min at room temperature before being added to the cells that were incubated for 72 h before fixation or lysis. For immunofluorescence assay, cells were transfected with calcium phosphate to introduce either pcDNA3.0 or RNF13WT-HA 48 h after DsiRNA transfection (24 h prior fixation).

## 3. Results

### 3.1. RNF13 Variants L311S and L312P Show Reduced Presence in Lysosomes and Alter Endosomal Vesicle Size

**Figure 1:**
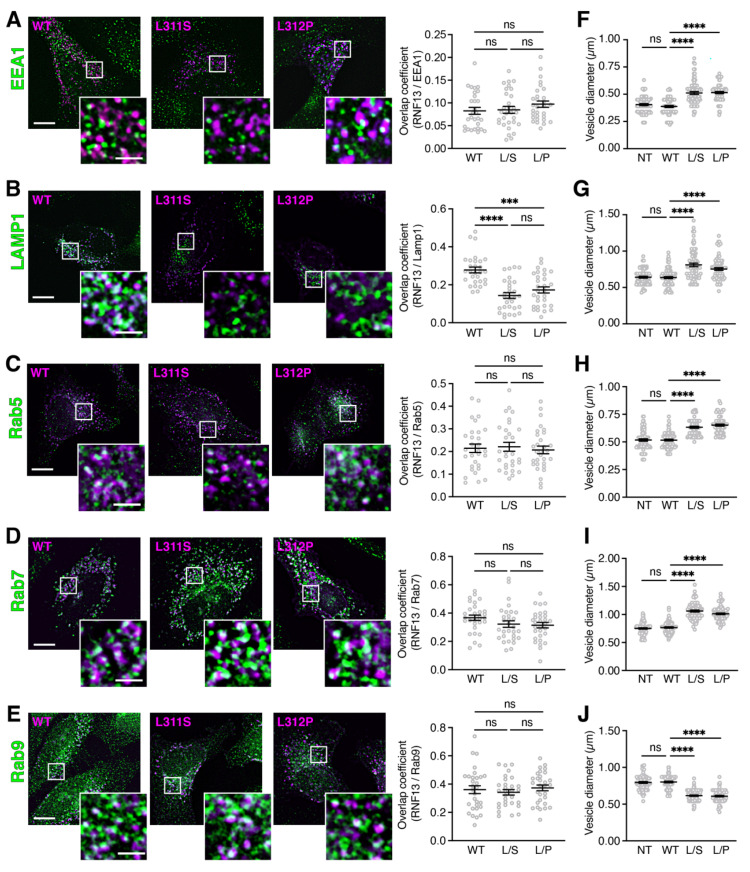
RNF13 L311S and L312P variants alter the size of endolysosomal vesicles. (**A**–**E**) Plasmid constructs encoding HA-tagged WT, L311S, or L312P RNF13 were transiently expressed in HeLa cells. Cells were fixed, permeabilized, and labeled with specific primary antibody against HA (RNF13) and against the endogenous subcellular markers. Representative images from three independent experiments (N = 3) show RNF13-HA (in purple) and (**A**) early endosome marker EEA1, (**B**) lysosome marker Lamp1, (**C**) early endosome marker Rab5, (**D**) late endosome marker Rab7, and (**E**) recycling endosome marker Rab9 (in green). Scale bars indicate 10 µm for whole cell image and 2 µm for the boxed area shown at higher magnification. The jittered individual value plots (**A**–**E**) represent Manders overlap coefficient of RNF13 over (**A**) EEA1, (**B**) Lamp1, (**C**) Rab5, (**D**) Rab7, or (**E**) Rab9. For each condition analyzed: N = 3, n = 30 cells. Values are expressed as mean ± SEM. Not significant (ns), *** *p* = 0.0004 or **** *p* < 0.0001 using one-way ANOVA with the Kruskal–Wallis test. The jitter strip plots ((**F**), EEA1; (**G**), Lamp1; (**H**), Rab5; (**I**), Rab7; (**J**)**,** Rab9) represent the diameter of the five largest vesicles for each cell. For each condition analyzed: N = 3, n = 15 cells, 75 vesicles. Values are expressed as mean ± SEM. Not significant (ns) or **** *p* < 0.0001 using one-way ANOVA with the Kruskal–Wallis test.

An altered cellular function for RNF13 variants L311S and L312P has been reported but a possible effect on their intracellular localization has not been investigated [40]. Because RNF13 is a known vesicular protein that localizes in endolysosomal compartments [23,28,43], we used immunofluorescence to investigate whether RNF13 WT is located in the endolysosomal system and if RNF13 L311S and L312P variant proteins retain a similar localization. To resolve the endosomal localization of the membrane protein RNF13, we transfected HeLa cells with HA-tagged RNF13 and immunostained for endogenous endosomal markers. First, using the early endosome marker Early Endosome Antigen 1 (EEA1), our results show that RNF13 WT and its variants L311S and L312P do not extensively colocalize with EEA1, as measured using Manders overlap coefficient (Figure 1A). In contrast, we observed and measured a significant overlap between RNF13 WT and the lysosomal-associated membrane protein 1 (Lamp1) (Figure 1B). Additionally, a significant reduction in colocalization between both L311S and L312P with Lamp1 was measured when compared to WT RNF13 with Lamp1 (Figure 1B; *** *p* = 0.0004 and **** *p* < 0.0001; see Table S1 for all statistical values), therefore suggesting that the lysosomal localization of RNF13 variants is altered.

We further investigated the RNF13 endolysosomal location using the Rab family of small GTPases. These are widely used as subcellular markers since distinct members of the family localize to different compartments to control the specificity and directionality of trafficking pathways [44]. For instance, Rab5 is specific to early endosomes [45], Rab7 is a late endosome marker [45], and Rab9 mediates vesicle transport from late endosomes to the trans-Golgi network (TGN) [46]. Our results show that RNF13, WT and its variants L311S and L312P, partially overlap with Rab5-positive vesicles (Figure 1C), Rab7-positive vesicles (Figure 1D), and Rab9-positive vesicles (Figure 1E). Manders overlap coefficients measured with regard to a specific Rab marker were not statistically different between RNF13 WT and its variant proteins (Figure 1C–E; Table S1). Because RNF13 is described also as an ER protein [29,30], the localization of HA-tagged human RNF13 L311S and L312P proteins was compared to that of RNF13 WT in HeLa cells co-labeled with protein disulfide-isomerase (PDI), a marker for endogenous ER-localized proteins. The results demonstrate limited colocalization between PDI and RNF13, suggesting that HA-tagged WT RNF13 and its L311S and L312P variant proteins do not extensively localize to the ER (Figure S1A).

During our analyses regarding the intracellular distribution of RNF13, we noticed that some vesicles appeared to be larger when cells were expressing RNF13 L311S or L312P compared to WT. Therefore, we measured the diameter of the largest vesicles positive for various markers of the endolysosomal pathway. Importantly, due to previous reports showing that ectopic expression of RNF13 WT can causes various cellular phenotypes [23,32,37], we also assessed the vesicle diameter in non-transfected cells (NTs). We found that the RNF13 L311S and L312P protein variants significantly increased the size of EEA1-positive vesicles by approximately 20% when compared to WT (Figure 1F; **** *p* < 0.0001; Table S2). A similar trend was found with Lamp1-positive vesicles (Figure 1G, **** *p* < 0.0001), Rab5-positive vesicles (Figure 1H; **** *p* < 0.0001), and Rab7-positive vesicles (Figure 1I; **** *p* < 0.0001). Conversely, the presence of RNF13 L311S or L312P variants significantly decreased the diameter of Rab9-positive vesicles by approximately 25% (Figure 1J; **** *p* < 0.0001; Table S2). Overall, our results show no significant difference between NT and WT RNF13 for every marker (Figure 1 F–J; ns *p* > 0.9999), whereas both RNF13 variants L311S and L312P significantly alter the size of vesicles belonging to the endolysosomal system.

### 3.2. RNF13 Variants L311S and L312P alter EGF Sorting at Early Endosomes

**Figure 2:**
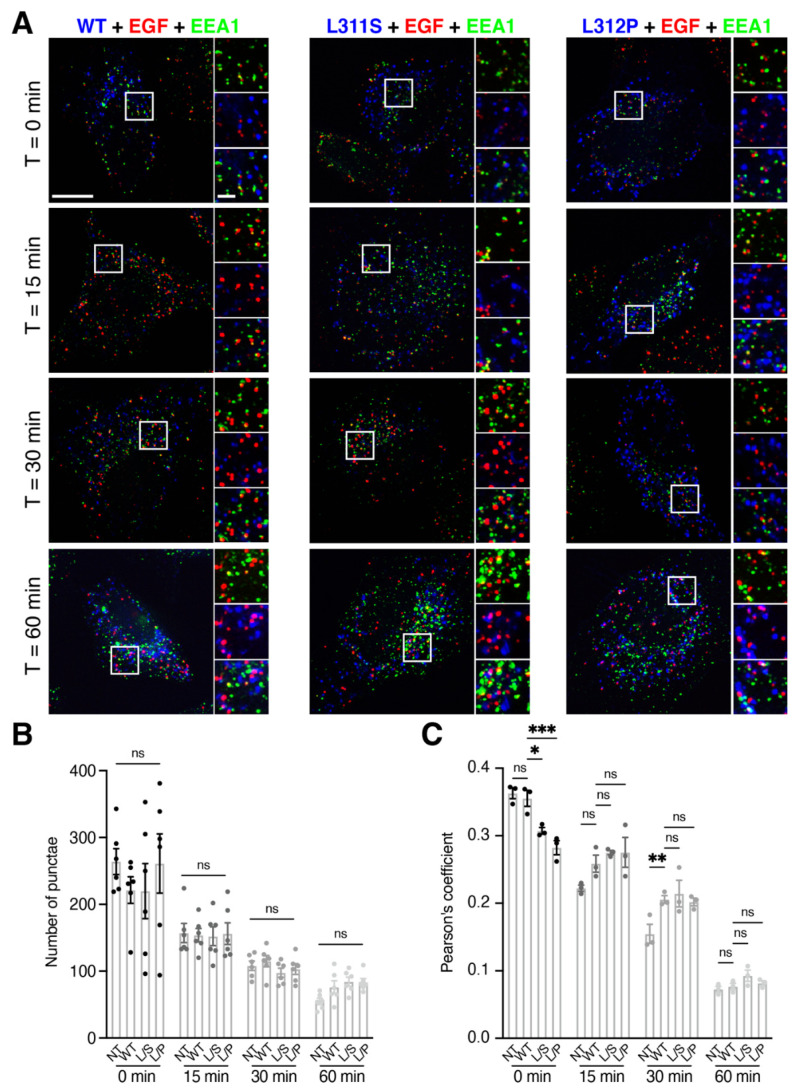
RNF13 variants L311S and L312P alter EGF sorting at early endosomes. (**A**–**C**) Starved HeLa cells transiently expressing RNF13-HA WT, L311S, or L312P were incubated with EGF for 5 min. Live cells were washed and incubated in fresh culture medium for the indicated time before being fixed, permeabilized, and labeled with specific primary antibody against HA (RNF13) and endogenous EEA1. (**A**) Representative images show EGF (red) with RNF13 (blue) and early endosome marker EEA1 (green). Scale bar = 10 µm. Boxed areas are shown at higher magnification (scale bar = 2 µm). (**B**) Quantitation of the average number of EGF puncta per cell. The bar graph presents the mean ± SEM while the strip plot represents the distribution of the average number of EGF puncta for each independent experiment. Analyses were performed on 6–9 cells within 5 fields of view collected for each of the six independent experiments (N = 6, total of 41 to 50 cells per condition). Not significant (ns) using two-way ANOVA with multiple comparison. (**C**) Quantitation of overlap between EGF and EEA1. The bar graph presents the mean ± SEM while the strip plot represents the distribution of the averaged Pearson’s coefficient of each independent experiment analyzed (N = 3). Each condition contains 37 to 45 cells. Not significant (ns), * *p* = 0.0155, ** *p* = 0.0093 or *** *p* = 0.0002 using two-way ANOVA with multiple comparisons.

Our findings suggest that RNF13 variants affect the endolysosomal system. The EGF receptor (EGFR) is a useful tool to study the trafficking and dynamics of the endolysosomal system. Upon EGF stimulation, EGFR is rapidly internalized and trafficked to be degraded in lysosomes [47,48]. To determine whether EGFR trafficking is altered by RNF13 protein variants, we performed a ligand-based EGF pulse-chase endocytosis experiment in transfected HeLa cells. Following a 5 min pulse with fluorescent EGF complex, a chase period was performed for up to 60 min. By using fluorescence microscopy, EGF and EEA1 fluorescence signals were obtained to track the progression of EGF in the endolysosomal system (Figure 2A). First, to evaluate the degradation of EGF over time, we analyzed the number of EGF puncta remaining after each timepoint. When HeLa cells expressed RNF13 WT, the number of puncta gradually decreased over time (Figure 2B; Table S3). Similar results were also obtained for RNF13 L311S, RNF13 L312P, and NT control cells (Figure 2B; Table S3) with no significant difference when compared to WT. These results suggest that the degradation rate of EGF remains similar for all the conditions. Thus, we wondered if the progression of EGF in the endolysosomal system was also similar. We determined the Pearson’s coefficient by analyzing the colocalization between EGF and EEA1 for each condition and timepoint (Figure 2C). For RNF13 WT, the Pearson’s coefficient gradually decreased over time (Figure 2C; Table S4). Interestingly, a significant difference was observed at t = 0 for both RNF13 L311S and RNF13 L312P when compared to WT, while NT was not significantly different (Table S4). While there was no difference observed at 15 min between all the conditions (Figure 2C), we measured a significant difference between NT and WT at 30 min while there was no significant difference between WT and the variants (Table S4). At 60 min, no difference was found between the conditions (Figure 2C; Table S4). Together, these results suggest that RNF13 variants L311S and L312P alter EGF trafficking at the early endosomes but do not affect the overall degradation.

### 3.3. RNF13 Variants L311S and L312P Do Not Alter Transferrin Trafficking

As described above, the variants L311S and L312P alter EGF sorting at the early endosomes. To test if other receptor trafficking may also be affected by the presence of RNF13 variants, we explored transferrin (Tf) uptake and trafficking. Similar to EGFR, Tf receptor (TfR) trafficking is a well-known and useful means to study the dynamics of the recycling pathway. Upon stimulation with Tf, TfR are internalized and sent to the early endosomes where they deliver iron before being recycled back to the plasma membrane [49]. To determine if RNF13 protein variants affect Tf trafficking, transfected HeLa cells were starved and briefly incubated with fluorescent human Tf before allowing internalization of the fluorescent Tf cargo for a period up to 60 min (Figure S2). Similar to the EGF analysis (Figure 2), endogenous labelling of EEA1 was performed to track the progression of Tf within the endolysosomal system over time. Our results show that the number of Tf puncta decreased without a significant change for the first 30 min in cells expressing L311S and L312P compared to WT (Figure S2A,B; Table S5). After 60 min, there were almost no detectable Tf puncta remaining in NT cells or in cells expressing RNF13 WT, L311S, or L312P (Figure S2A,B; Table S5). These results suggest that RNF13 L311S and L312P do not affect Tf recycling. To evaluate the progression of Tf in the endolysosomal system, we analyzed the colocalization between Tf and EEA1 for each condition (Figure S2C; Table S6). The measured Pearson’s coefficients slowly decreased over time but did not significantly change between each timepoint in any of the four conditions (Figure S2C; Table S6). Overall, our results suggest that the RNF13 genetic variants L311S and L312P do not affect Tf trafficking.

### 3.4. RNF13 Variant L311L312/AA Shows Reduced Presence in Lysosomes and Increases Endosomal Vesicle Size

**Figure 3:**
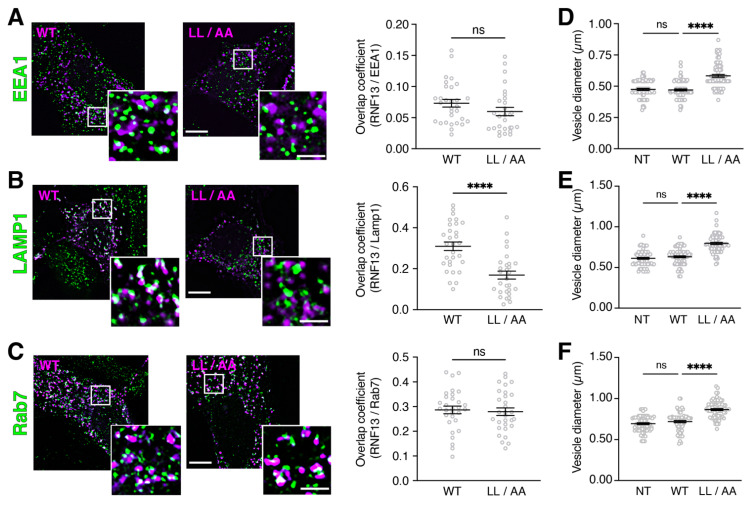
Altered lysosomal targeting of RNF13 L311L312/AA mutant increases the size of endolysosomal vesicles. (**A**–**C**) Plasmid constructs encoding HA-tagged RNF13 WT or LL/AA were transiently expressed in HeLa cells. Cells were fixed, permeabilized, and labeled with specific primary antibody against HA (RNF13) and against the endogenous subcellular markers. Representative images from three independent experiments (N = 3) show RNF13-HA (in purple) and (**A**) early endosome marker EEA1, (**B**) lysosome marker Lamp1, and (**C**) late endosome marker Rab7 (in green). Scale bars indicate 10 µm for whole cell image and 2 µm for the boxed area shown at higher magnification. The jittered individual value plots (**A**–**C**) represent Manders overlap coefficient of RNF13 over (**A**) EEA1, (**B**) Lamp1, and (**C**) Rab7. For each condition analyzed: N = 3, n = 30 cells. Values are expressed as mean ± SEM. Not significant (ns) or **** *p* < 0.0001 using the Mann–Whitney test. (**D**–**F**) The jittered individual value plots ((**D**), EEA1; (**E**), Lamp1; (**F**), Rab7) represent the diameter of the five largest vesicles for each cell. For each condition analyzed: N = 3, n = 15 cells, 75 vesicles. Values are expressed as mean ± SEM. Not significant (ns) or **** *p* < 0.0001 using one-way ANOVA with the Kruskal–Wallis test.

RNF13 variants L311S and L312P change the dileucine motif of the WT protein [40]. The WT RNF13 307-EHTPLL-312 motif resembles a canonical [D/E]xxxL[L/I]-type dileucine sorting signal known for binding cellular adaptor protein (AP) complexes and appropriately routing several transmembrane proteins, including vesicle-associated membrane protein 4 (VAMP4), cluster of differentiation 4 (CD4), and vesicular monoamine transporter 1 (VMAT1) [50]. Since classical examination of the roles of dileucine motifs in membrane protein trafficking usually relies on the replacement of the hydrophobic dileucine residues with alanines to obtain a non-functional motif [50], we therefore generated the AP-binding defective RNF13 mutant L311L312/AA (hereafter identified as LL/AA). First, we examined whether the RNF13 LL/AA mutant phenotypically behaves similarly to L311S and L312P variants that, as shown in Figure 1, exhibit an altered lysosomal localization while increasing endolysosomal vesicle size. Our results show that WT or LL/AA proteins do not overlap with EEA1 in transfected HeLa cells (Figure 3A; Table S7). We observed that the RNF13 LL/AA exhibits a diminished overlap with Lamp1 as compared with RNF13 WT (Figure 3B, **** *p* < 0.0001; Table S7). Additionally, late endosomal marker Rab7 overlaps with both WT and LL/AA (Figure 3C; Table S7), a result highly similar to the variants L311S and L312P (Figure 1D). To assess the impact of RNF13 LL/AA on endolysosomal vesicle morphology, we measured the diameter of the largest vesicles and found that RNF13 LL/AA significantly increased the size of EEA1-positive vesicles (Figure 3D; **** *p* < 0.0001; Table S8), Lamp1-positive vesicles (Figure 3E; **** *p* < 0.0001; Table S8), and Rab7-positive vesicles (Figure 3F; **** *p* < 0.0001; Table S8). Once again, no difference was observed between overexpressed RNF13 WT and NT cells (Figure 3D,F; Table S8). Overall, our results show that RNF13 LL/AA recapitulates the cellular phenotypes of L311S and L312P variants and support that RNF13 having an altered EHTPLL dileucine motif cannot be properly localized to lysosomes.

### 3.5. RNF13 Variants L311S and L312P Do Not Interact with AP-3 Complex

**Figure 4:**
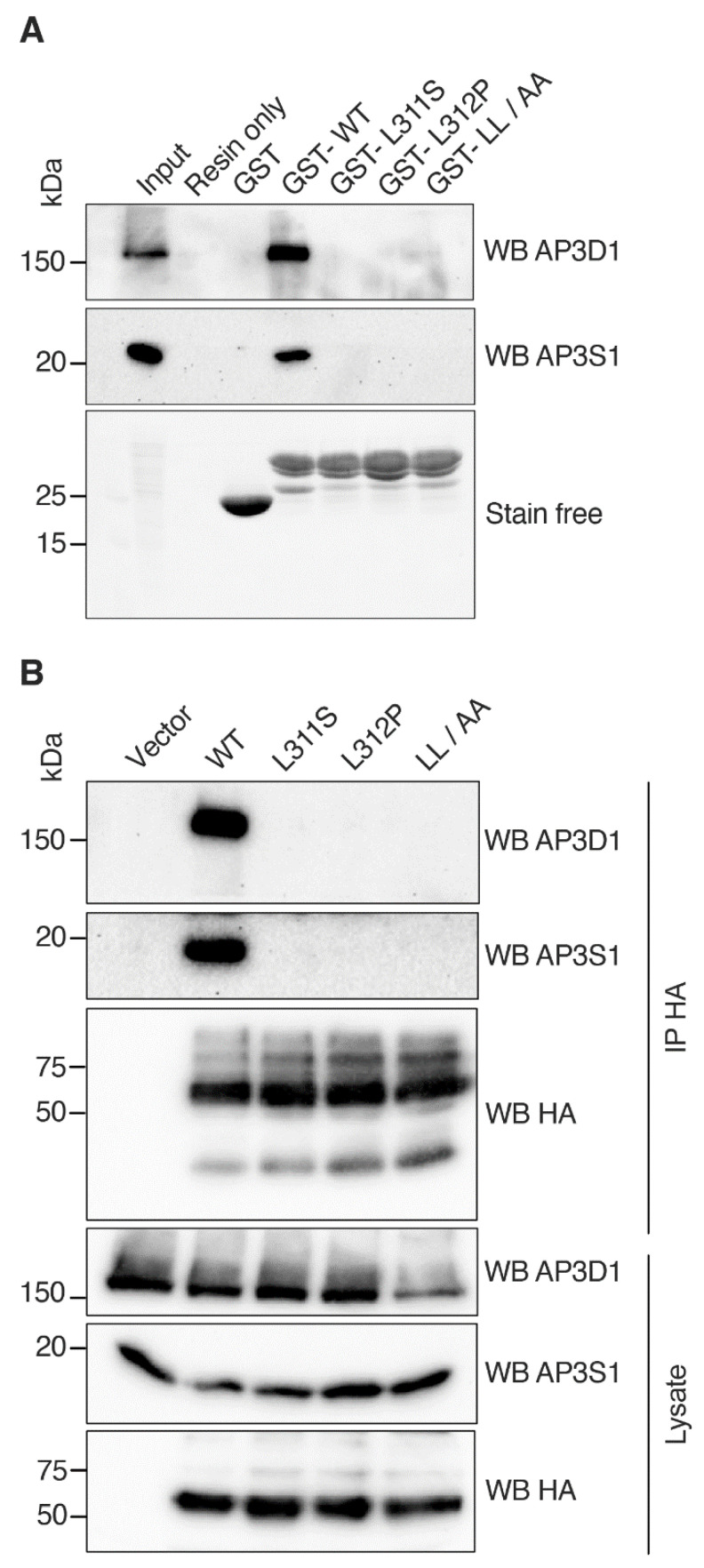
Alteration of the dileucine motif of RNF13 abrogates AP-3 complex binding. (**A**) GST pulldown was performed with HEK293T cell lysate incubated in the presence or absence of GST or purified GST-RNF13 (aa 281-381; WT, L311S, L312P, or LL/AA) immobilized on glutathione-functionalized sepharose 4B affinity resin. Complex formation was performed overnight at 4 °C, followed by washes and elution in 2× Laemmli buffer. Proteins were separated on SDS–PAGE and transferred to a PVDF membrane which was immunoblotted using specific primary antibodies against AP3S1 and AP3D1. Representative immunoblots from three independent experiments (N = 3) are shown. (**B**) Plasmid constructs encoding HA-tagged WT, L311S, L312P, or L311L312/AA RNF13 were transiently expressed in HEK293T cells. Following lysis, cell lysates were incubated for 2 h at 4 °C and then anti-HA immunoprecipitated complexes were washed and eluted in 2× Laemmli buffer. Proteins were separated on SDS–PAGE and transferred to a PVDF membrane which was immunoblotted using specific primary antibodies against AP3S1, AP3D1, and HA (RNF13). Representative immunoblots from three independent experiments (N = 3) are shown.

Thus far, our results demonstrate that an intact dileucine motif within RNF13 is required for its appropriate lysosomal localization. Because intact dileucine motifs are critical for binding AP complexes, we hypothesized that RNF13 would bind to AP complex and that dileucine variants, either L311S, L312P, or LL/AA, would greatly reduce if not completely abrogate the interaction with AP complexes. Amongst the five AP complexes that exist, only AP-1, AP-2, and AP-3 appear to recognize dileucine-based ([D/E]xxxL[L/I]) signals [51]. Given that AP-3, which is responsible for transport from tubular endosomes to late endosomes and for the biogenesis of lysosome-related organelles [51], has been recently identified by mass spectrometry analyses to bind RNF13 [52,53], we thus investigated whether RNF13 variants L311S and L312P can associate with AP-3. First, in a GST pulldown assay, endogenous AP-3 complexes found in HEK293T cell lysate were incubated with purified GST-fused RNF13 intracellular domain (amino acids 281–381) proteins immobilized on glutathione-functionalized sepharose 4B affinity resin. AP-3 complex binding to RNF13 WT was compared to variants L311S and L312P, and to the anticipated RNF13 AP-binding defective LL/AA mutant. Specific immunoblotting of GST pulldown assay samples revealed that AP3S1 and AP3D1 subunits of the AP-3 complex bind to RNF13 WT (Figure 4A). In contrast, AP-3 subunits do not interact with either RNF13 LL/AA or the variants L311S and L312P (Figure 4A). Next, we assessed whether RNF13 binds AP-3 complex in a cellular context by expressing HA-tagged RNF13 WT, L311S, L312P, or LL/AA in HEK293T cells. HA-tagged RNF13 proteins were immunoprecipitated with an anti-HA before the presence of AP-3 complex in the immunopurified proteins was determined using AP3S1 and AP3D1 antibodies. The results show that RNF13 WT binds to AP-3 complex while all the variants fail to do so (Figure 4B). Together, those results demonstrate that the tested mutations in RNF13 dileucine motif alter interaction with the AP-3 complex.

### 3.6. Model of RNF13 and AP3S1 Complex Association

**Figure 5:**
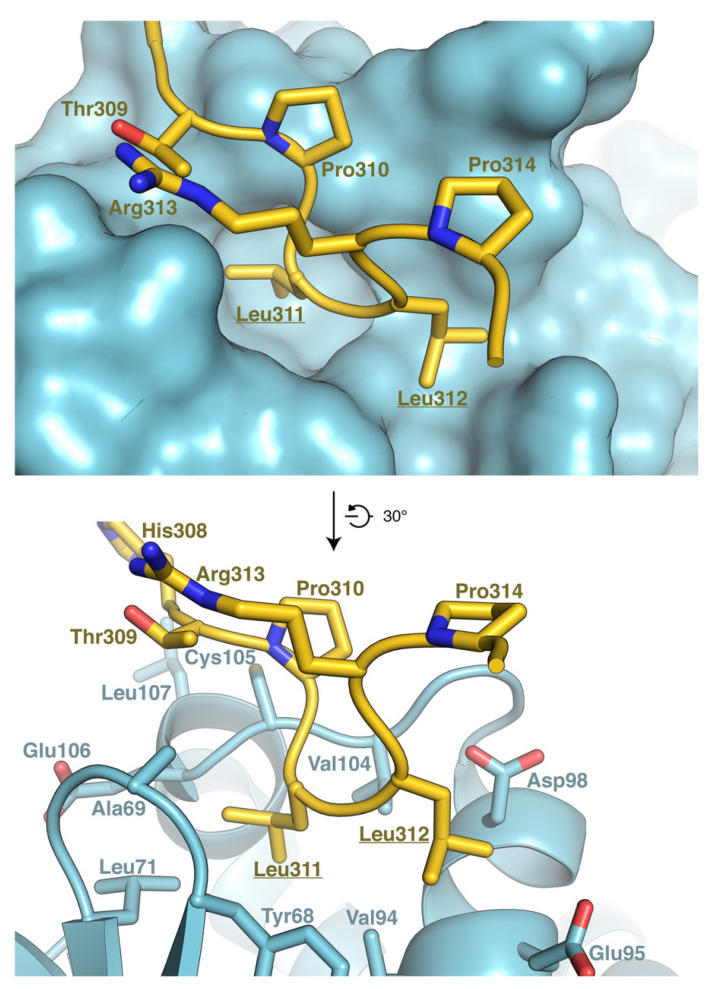
Predicted structure of a complex between AP3S1 and RNF13. The interaction between RNF13 in yellow and AP3S1 in blue was predicted using AlphaFold. Top panel shows RNF13 in sticks and AP3S1 is surface representation. Bottom panel shows RNF13 and AP3S1 in stick representation. In both panels, the two leucine residues which are mutated in DEE73 are underlined. The residues of RNF13 that do not interact with AP3S1 were omitted from the figure for clarity.

To better understand the importance of the dileucine motif for RNF13–AP3S1 complex formation, we generated a model of the complex using AlphaFold (Figure 5). Our model shows that the 307-ETHPLL-312 motif of RNF13 interacts with AP3S1. Specifically, the dileucine motif of RNF13 is inserted in a hydrophobic cavity of AP3S1, and interacts with AP3S1 residues Val105, Leu71, Tyr68, and Val94, as well as with the aliphatic portions of Glu95 and Asp98 (Figure 5). Leu311 of RNF13 seems to be especially important for the complex since this residue is located in a deeper hydrophobic pocket of AP3S1 (Figure 5). In the case of RNF13 Leu312, although it is close to polar residues, it mostly interacts with the hydrophobic regions of these residues (Figure 5). Our model also shows that the loop containing the dileucine motif is maintained in a configuration suitable for AP3S1 interaction through hydrogen bonding between Thr309 and Arg313 and through hydrophobic packing between Pro310 and the aliphatic portion of Arg313 (Figure 5). Overall, this analysis illustrates the importance of the dileucine motif and neighboring residues in the interaction between RNF13 and AP3S1.

### 3.7. Knockdown of AP-3 Gene Expressions Reduces RNF13 Localization to Lysosomes

**Figure 6:**
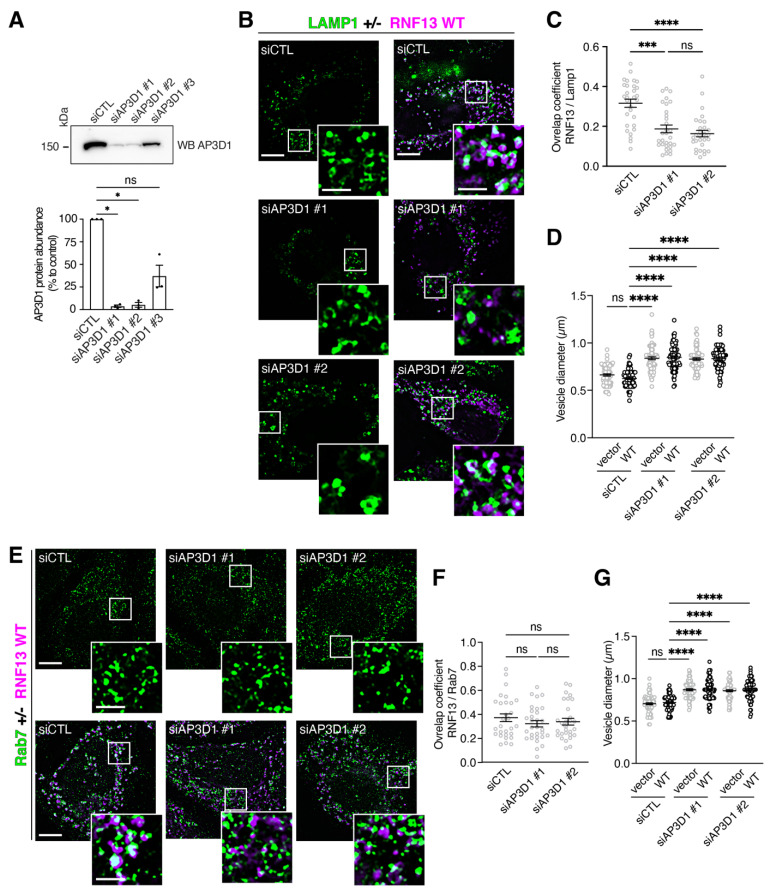
Knockdown of AP-3 interferes with RNF13 localization to lysosomes. (**A**) HeLa cells were transfected using 10 nM of Dicer-substrate siRNAs (DsiRNAs) specific against AP3D1 or nontargeting (siCTL). Three DsiRNAs specific against AP3D1 were tested. Seventy-two hours after transfection, cells were lysed and diluted in Laemmli buffer. Proteins were separated on SDS–PAGE and transferred to a PVDF membrane which was immunoblotted using specific primary antibody against AP3D1. Band densities were analyzed and normalized to the band density of the siCTL lane using ImageLab software. The bar graph presents the mean ± SEM while the strip plot represents the averaged relative protein abundance of each independent experiment analyzed (N = 3). Not significant (ns) or * *p* < 0.05 using one-way ANOVA with the Kruskal–Wallis test. (**B**–**G**) HeLa were transfected using 10 nM of DsiRNAs #1, #2 or siCTL for 72 h. Twenty-four hours prior to fixation, cells were transfected with either a plasmid construct encoding HA-tagged RNF13WT or an empty vector. Cells were fixed, permeabilized, and labeled with specific primary antibody against HA (RNF13) and against the endogenous subcellular markers. Representative images from three independent experiments (N = 3) show RNF13-HA (in purple) and (**B**) lysosome marker Lamp1 and (**E**) late endosome marker Rab7 (in green). Scale bars indicate 10 µm for whole-cell image and 2 µm for the boxed area shown at higher magnification. The jittered individual value plots (**C**,**F**) represent the Manders coefficient of RNF13 over (**C**) Lamp1 or (**F**) Rab7. For each condition analyzed: N = 3, n = 30 cells. Values are expressed as mean ± SEM. Not significant (ns), *** *p* = 0.0001 or **** *p* < 0.0001 using one-way ANOVA with the Kruskal–Wallis test. The jittered individual value plots (**D,G**) represent the diameter of the five largest vesicles for each cell. For each condition analyzed: N = 3, n = 15 cells, 75 vesicles. Values are expressed as mean ± SEM. Not significant (ns) or **** *p* < 0.0001 using one-way ANOVA with the Kruskal–Wallis test.

Based on our findings, the RNF13/AP-3 complex association appears to regulate the targeting of RNF13 to lysosomes. As AP-3 plays a role in trafficking membrane protein to lysosomes [51], we speculate that AP-3 might regulate the lysosomal localization of RNF13. To investigate the role of endogenous AP-3 expression on RNF13, we used Dicer-substrate siRNA (DsiRNA) duplexes to knockdown the delta subunit of AP-3 (AP3D1). After HeLa cells were transfected with a nontargeting control (CTL) and three DsiRNA against AP3D1 for 72 h, cell lysate proteins were probed with specific antibody (Figure 6A). We found that AP3D1 relative protein abundance significantly declined with DsiRNAs #1 and #2 but not with DsiRNA #3 (Figure 6A; CTL: 100%; siAP3D1 #1: 3.723% ± 1.337%; * *p* = 0.0262; siAP3D1 #2: 5.264% ± 2.589%; * *p* = 0.0364; siAP3D1 #3: 37.13% ± 11.99%; ns *p* = 0.9143). We then characterized the impact of reducing AP3D1 expression on RNF13 lysosomal localization and vesicle morphology. First, Lamp1 and Rab7 exhibit similar distribution and fluorescence intensity in the presence of siAP3D1 when compared to siCTL (Figure 6B,E). These results show that AP-3 knockdown does not affect the markers for our vesicles of interest. However, while RNF13 WT overlaps greatly with Lamp1 in control conditions (Figure 6B,C), AP3D1 knockdown using both siAP3D1 #1 and #2 extensively reduce RNF13 WT and Lamp1 colocalization (Figure 6B,C). These observations were confirmed using the Manders coefficients, which display a significant reduction in overlapping between RNF13 WT and Lamp1 in the presence of siAP3D1 #1 and #2 when compared to control (Figure 6C; *** *p* = 0.0001, **** *p* < 0.0001; Table S9). We next assessed the impact of AP-3 knockdown on endolysosomal vesicle morphology by measuring the diameter of the largest vesicles positive for both Lamp1 and found that siAP3D1 #1 and #2 significantly increased the size of Lamp1-positive vesicles independently of RNF13 WT presence or absence when compared to siCTL (Figure 6D; Table S10). When investigating the effect of abrogating AP3D1 expression with the late endosomal marker Rab7, we found that RNF13 WT overlaps with Rab7 in control and AP3D1 depleted cells and that there is no difference in Manders overlap coefficients for each condition when compared to control (Figure 6E,F; Table S9). Finally, our results show that AP3D1 knockdown significantly increased the size of Rab7-positive vesicles independently of RNF13 WT presence or absence when compared to siCTL (Figure 6G; Table S10). Overall, our results suggest that RNF13 targeting to lysosomes depends on its association with the AP-3 complex, and that this association is critical for endolysosomal vesicle size.

## 4. Discussion

Eukaryotic cells use several intracellular compartments where resident proteins must be appropriately directed for normal organelle function. Aberrant location and targeting of a protein can be caused by mutation, altered expression of cargo proteins, or disruption of the routing machinery, all of which can lead to protein inactivation, loss of protein–protein interaction, or harmful activity in the wrong compartment [54,55]. A recent study reported two missense genetic mutations in the gene encoding the ubiquitin ligase RNF13, resulting in protein variants L311S and L312P [40]. The lack of molecular understanding regarding the biological impact of RNF13 variants prompted us to better define the cellular mechanisms associated with RNF13. By using a fluorescence microscopy approach, the present study shows that WT RNF13 is localized to endosomes and lysosomes, while the presence of RNF13 variant proteins L311S and L312P in lysosomes is reduced. Furthermore, our results show that RNF13 variant proteins L311S and L312P affect the morphology of endosomal vesicles as well as the temporal and spatial progression of fluorescent EGF but not Tf cargos in the endolysosomal system. Interestingly, our results demonstrate that AP-3 complex interaction with RNF13 variants L311S and L312P, as well as the RNF13 AP-binding defective variant LL/AA, is abrogated, whereas the tested RNF13 variant proteins similarly affect vesicle size and lysosomal localization. Finally, knockdown of the AP3D1 subunit leads to a reduced presence of RNF13 WT in lysosomes and a similar alteration of the morphology of endosomal vesicles, as observed with the RNF13 variants.

Our analyses of endolysosomal vesicles exposed significant differences between RNF13 WT and the variants. Indeed, significantly smaller Rab9-positive vesicles were found with L311S and L312P variants, whereas Rab5-, Rab7-, EEA1- and Lamp1-positive vesicles were significantly enlarged. Over the years, multiple mechanisms have been shown to produce enlarged endosomes. For instance, it is known that a defect in endosome maturation, such as the disruption of the Rab5-to-Rab7 switch, results in enlarged early endosomes [56,57,58]. Interestingly, overexpression of Rab5 or expression of a constitutively active mutant, Rab5Q79L, causes enlarged endosomes by accelerating the rate of endocytosis [59,60]. However, some studies have shown that even in the presence of enlarged early endosomes, Tf trafficking is not necessarily altered [56,61,62]. Concordantly, in our study, even though early endosomes are enlarged in cells expressing RNF13 L311S and L312P, trafficking of Tf is not altered. Furthermore, EGF degradation is similar between WT- and variant-expressing cells. Based on the colocalization of EEA1 with EGF, but not Tf, EGF seems to pass through early endosomes of L311S- and L312P-expressing cells at a different rate when compared to WT. The significance of this result requires further investigation. Our analyses also demonstrate that a similar amount of EGF remains at both 30 and 60 min, implying that the late endocytic pathway retains efficient trafficking/sorting of endocytosed cargo. In this context, if the endocytic rate is altered with the RNF13 L311S and L312P variants but EGF degradation is similar for the variants and WT RNF13, this may reveal an altered function of the enlarged late endosomes/lysosomes.

In this study, RNF13 WT primarily overlaps with endolysosomal markers, as previously reported elsewhere [23,28,43], in contrast with RNF13 being described in the ER [23,29,30]. While we are unsure of the reason for the discrepancy between the reported ER and endolysosomal localizations, a possible explanation could be the use of different constructs. Specifically, since RNF13 has a signal peptide on the N-terminus that is needed for its proper biosynthesis, we positioned a HA tag at the C-terminus, which rarely interferes with the normal function and distribution of proteins [63]. Because we did not find a suitable antibody to verify the intracellular localization of endogenous RNF13, we used a GFP-fused protein to test whether both constructs would give similar results regarding intracellular localization. Under our tested conditions, the overall vesicle-looking localization of HA-tagged RNF13 protein was indiscernible from RNF13-GFP (Figure S3). Another putative explanation for the discrepancy between our results consistently showing an endolysosomal localization for RNF13 rather than an ER localization could be the use of different cell lines. In fact, HeLa cells were previously used as a study model, where the authors reported that RNF13 possesses an endolysosomal localization [28,43], which is fully consistent with our study. Regarding the RNF13 L311S and L312P variants, they did not mislocalize, at steady-state, to the ER or the Golgi, suggesting that variant proteins are likely trafficked adequately within these organelles of the biosynthetic pathway (Figure S1). However, knowing that ER stress responsiveness and apoptosis signaling are two functions previously reported for RNF13 [40], we speculated that RNF13 WT and L311S and L312P variants could accumulate in the ER when cells are treated with ER stress-inducing drugs [30]. After using the ER Ca^2+^–ATPase inhibitor, thapsigargin, or the N-glycosylation inhibitor, tunicamycin, we did not detect an increased accumulation of RNF13 WT or the variant proteins in the ER (Figure S4). Therefore, it would be rather surprising if the RNF13 L311S and L312P variant proteins increased apoptosis through ER accumulation. However, since mounting evidence suggests that organelles, such as the ER, the lysosomes, and the Golgi apparatus, are major integrators of pro-apoptotic signaling [64], our results cannot exclude that RNF13 variants may be facilitating, from an endosomal location, apoptosis signaling, as observed in L311S-expressing patient-derived cells [40].

Our study uncovered that RNF13 L311S and L312P variants enlarge lysosomes in HeLa cells. Enlarged lysosomes are observed in a variety of diseases, such as Charcot–Marie–Tooth, Chediak–Higashi syndrome, and lysosomal storage disorders (LSD), which include approximately 50 genetic diseases [65]. LSD are caused by deficiencies in lysosomal or non-lysosomal resident proteins. These deficiencies may be due to genetic alterations that disturb the synthesis or transport of lysosomal proteins and contribute to enlarged lysosomal structures [65]. Typically, enlarged lysosomes are caused by the accumulation of nondegradable material and have multiple impacts on other organelles and overall cell function [66]. However, enlarged lysosomes does not necessarily indicate an endocytic degradation dysfunction. Indeed, mutations in the lysosomal trafficking regulator protein (LYST) cause Chediak–Higashi syndrome, a rare autosomal recessive disease characterized by enlarged intracellular vesicles. A study conducted by Holland et al. (2014) showed that RNA depletion of LYST recapitulates the disease phenotype and, despite enlarged lysosomes, depletion of LYST protein does not affect normal rates of degradation [67]. Specifically, depletion of LYST protein did not affect EGF degradation and Tf trafficking, similarly to what we obtained with RNF13 variants L311S and L312P [67]. However, the accumulation of unmetabolized substrates in lysosomes can lead to alteration of lipid trafficking and calcium homeostasis or to increased oxidative stress, inflammation, autophagy, and/or ER stress [68]. Compatibly, cultured cells derived from patients with different types of LSD showed that apoptosis was induced by ER and oxidative stresses [69]. Based on our results and the hereabove cited studies, it is therefore highly plausible that endosomal RNF13 variant proteins L311S and L312P may alter lysosomal function and cellular homeostasis, leading to the modification of apoptotic signaling. Accordingly, this hypothesis would support the increase in apoptosis markers spliced XPB1 and phosphorylated c-Jun in cells derived from an individual carrying the L311S variant [40]. Future studies will be required to test this highly speculative molecular mechanism.

The AP complexes are heterotetrameric protein complexes that regulate intracellular trafficking [51]. Five AP complexes, known as AP-1, AP-2, AP-3, AP-4, and AP-5, are each composed of two large subunits (γ/α/δ/ε/ζ and β1-5, respectively), one medium-sized subunit (μ1-5), and one small-sized subunit (σ1-5), and each AP complex has a specific localization and function [51]. Consistent with the [D/E]xxxL[L/I]-type dileucine canonical sorting signal [40] being recognized by AP-1, AP-2, and AP-3 [51], our data support a model where RNF13 undergoes AP-dependent intracellular routing. AP-1 is associated with the trans-Golgi network and early and recycling endosomes, whereas AP-2 is mostly found at the plasma membrane [70]. Because our results with RNF13 variants L311S and L312P show a reduced presence in lysosomes, and based on the predicted model, we have exclusively studied the AP-3 complex, since this AP mediates transport from tubular endosomes to late endosomes and lysosomes and is involved in the biogenesis of lysosome-related organelles [51,70]. We demonstrate that the variants L311S and L312P alter the 307-EHTPLL-312 dileucine motif of RNF13 protein and consequently modify the intracellular distribution, likely due to a lost ability to bind AP-3, whereas the gene repression of AP-3 alters RNF13 targeting to lysosomes. In addition to AP-3, other AP complexes are expected to bind the dileucine motif of RNF13. Supporting this affirmation, recent studies used affinity purification and mass spectrometry analyses and found that the σ (isoform 2) subunit of AP-1, as well as the δ and σ (isoform 1 and 2) subunits of AP-3 complexes, bind to RNF13 [51,52,53]. Interestingly, the 470-DERAPLI-476 and 509-EEKQPLL-515 signals of lysosome membrane protein 2 (LIMP-II) and tyrosinase (TYR), respectively, bind to AP-3 but not AP-1 and AP-2, whereas motifs such as 2-DDQRDLI-8 and 11-NEQLPML-17 of the invariant chain (Ii) preferentially bind to AP-1 and AP-2 but not to AP-3 [50,71,72]. Interestingly, the interaction between the [D/E]xxxL[L/I] motif and specific AP complexes seem to be dictated by the “x” residues and both AP-3 interacting proteins LIMP-II and TYR have a proline in the -1 position of the first leucine [50]. In this context, our model shows that RNF13 proline at position 310 (Pro310) interacts with the arginine 313, thus strongly suggesting that Pro310 generates a favorable structure for binding the AP3S1 subunit of the AP-3 complex. Nevertheless, further studies will be required to validate not only the importance of the Pro310 for the formation of the RNF13–AP-3 complex, but also to investigate the function of the other residues within or surrounding the RNF13 dileucine motif.

An intriguing element in our study is the fact that the gene expression of the AP3 δ subunit reduces the presence of WT RNF13 in Lamp1-positive structures, whereas AP3D1 knockdown does not affect RNF13 presence in Rab7-positive vesicles. Accordingly, these results are supported by our data, showing that RNF13 variants are present in Rab7-positive vesicles without being in Lamp1-positive vesicles. Knowing that Rab7 is critical for controlling many aspects of endosomal dynamics such as the fusion of late endosomes with lysosomes, our data strongly suggest that the routing of RNF13 variants to late endosomes (Rab7-positive structures) occurs normally and through an AP-3-independent pathway, whereas RNF13 lysosomal localization requires AP-3 binding. To explain the proper late endosomal localization of RNF13, it is possible that RNF13 intracellular routing is similar to the NPC intracellular cholesterol transporter 1 (NPC1) protein. NPC1 predominantly localizes to late endosomes and has a C-terminal dileucine motif [73,74] that allows its late endosomal targeting through AP-3 binding [74]. Mutation of NPC1 dileucine motif leads to an increased trafficking via the plasma membrane, such that the NPC1 still ends up in its proper late endosomal location, suggesting that other signals contribute to NPC1 targeting [73]. It should be noted that the plasma membrane positioning of RNF13 was suggested previously [28] but the biological significance and the mechanism involved in the process remain currently unknown. Our study strongly supports that AP-3 has an important role in regulating RNF13 trafficking, but we cannot exclude that other signals within RNF13 and other molecular entities are contributing to RNF13 proper localization. Nonetheless, RNF13 presence in lysosomes seems to be important for proper endolysosomal vesicle morphology, because we observed a similar phenotype for all variants and AP-3 knockdown. It is possible that the modified binding of RNF13 L311S and L312P with the AP-3 complex alters endolysosomal structure and dynamics because of abnormal RNF13 trafficking to lysosomes, where it would normally achieve its cellular function.

## Data Availability

The data that support the findings of this study are available from the corresponding author upon reasonable request.

## Supplementary Materials

The following are available online at https://www.mdpi.com/article/10.3390/cells10113063/s1, Figure S1. RNF13 protein variants do not extensively localize in endoplasmic reticulum, mitochondria or Golgi compartments; Figure S2. RNF13 variants L311S and L312P alter transferrin recycling; Figure S3. Localization of RNF13 is not affected by the presence of either HA- or GFPtag; Figure S4. Chemically induced endoplasmic reticulum stress does not promote extensive ER localization of RNF13 in HeLa cells; Table S1: Statistical values-Manders overlap coefficient, Figure 1A–E; Table S2. Statistical values-Vesicle diameter, Figure 1F–J; Table S3. Statistical values-Number of punctae (EGF), Figure 2B; Table S4. Statistical values-Pearson’s coefficient (EGF), Figure 2C; Table S5. Statistical values-Number of punctae (Tf) Figure S2B; Table S6. Statistical values-Pearson’s coefficient (Tf), Figure S2C; Table S7. Statistical values–Manders overlap coefficient, Figure 3A–C; Table S8. Statistical values–Vesicle diameter, Figure 3D–F; Table S9. Statistical values–Manders overlap coefficient, Figure 6C,F; Table S10. Statistical values–Vesicle diameter, Figure 6D,G.

## Abbreviations

AIF, apoptosis-inducing factor; AP, adaptor protein; CTL, nontargeting control; DEE, developmental and epileptic encephalopathy; DsiRNA, dicer-substrate siRNA; EEA1, early endosome antigen 1; EDTA, ethylenediaminetetraacetic acid; EGF, epidermal growth factor; EGFR, epidermal growth factor receptor; ER, endoplasmic reticulum; Ii, invariant chain; IPTG, isopropyl-β-d-thiogalactoside; Lamp1, lysosomal-associated membrane protein 1; LIMP-II, lysosome membrane protein 2; LSD, lysosomal storage disorders; LYST, lysosomal trafficking regulator protein; NPC1, NPC intracellular cholesterol transporter 1; NT, non-transfected; PBS, phosphate-buffered saline; PMSF, phenylmethylsulfonyl fluoride; RCAS1, receptor-binding cancer antigen expressed in SiSo cells; RING, Really Interesting New Gene; RNF13, RING finger protein 13; TCE, 2,2,2-trichloroethanol; Tf, transferrin; TfR, transferrin receptor; Tg, thapsigargin; TGN, trans-Golgi network; Tn, tunicamycin; TYR, tyrosinase; WT, wild-type.
