# Supplementary material for: RNF13 Dileucine Motif Variants L311S and L312P Interfere with Endosomal Localization and AP-3 Complex Association
Source: Cells. 2021 Nov 6;10(11):3063. doi: 10.3390/cells10113063 (PMC8620429; doi:10.3390/cells10113063)
Supplement: Supplementary file 1 [file cells-10-03063-s001.zip › Cabana et al 2021_ supplementary figures S1-S4 final.pdf]

SUPPLEMENTARY FIGURES S1-S4

RNF13 dileucine motif variants L311S and L312P interfere with endosomal localization and AP-3 complex association

Valérie C. Cabana, Antoine Y. Bouchard, Audrey M. Sénécal, Kim Ghilarducci, Saïd Kourrich, Laurent Cappadocia, Marc P. Lussier

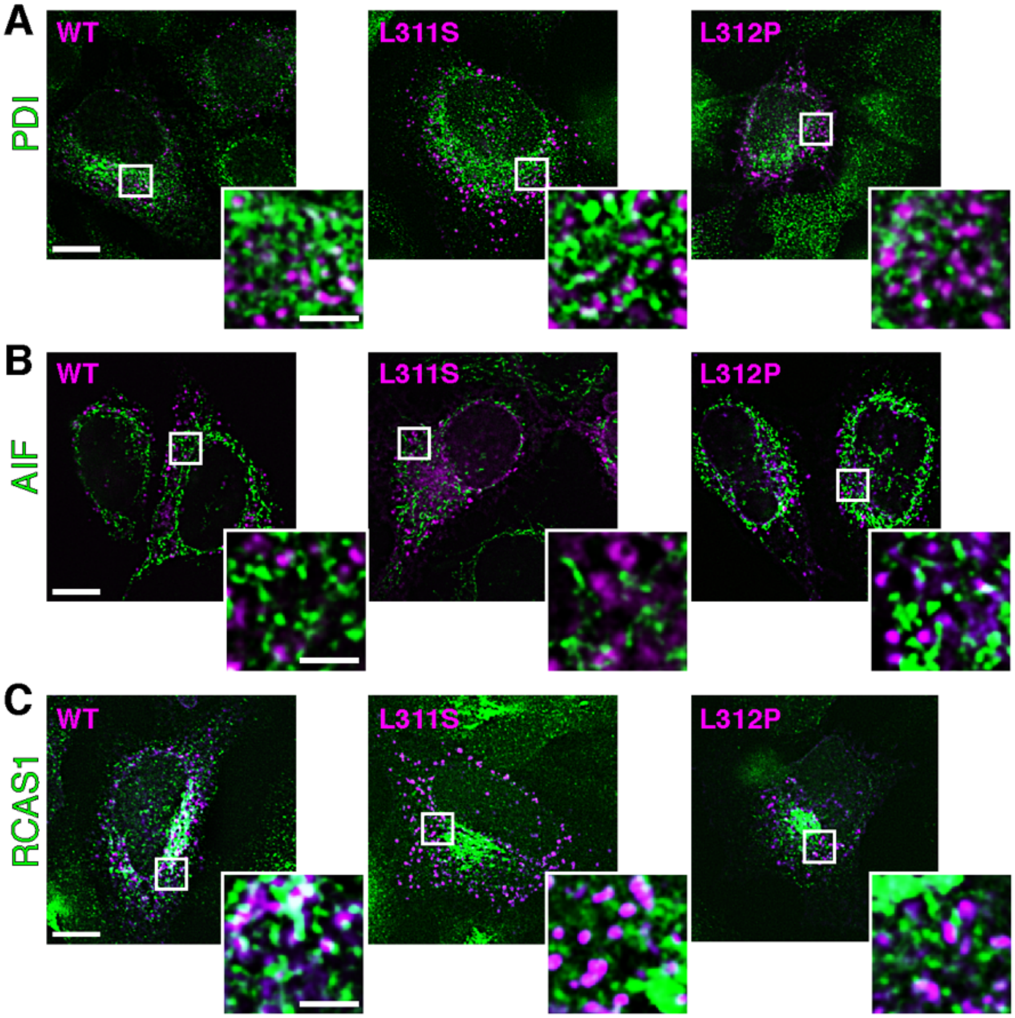

Figure S1. RNF13 protein variants do not extensively localize in endoplasmic reticulum, mitochondria or Golgi compartments. Using immunofluorescence microscopy, the localization of HA-tagged human RNF13 L311S and L312P proteins was compared to that of RNF13 WT in HeLa cells co-labeled various marker. (**S1A-C**) Representative fluorescence microscopy images from three independent experiments (N=3) show the subcellular distribution of HA-tagged RNF13, either wild-type (WT) or the L311S or L312P variants, transiently expressed in HeLa cells. Cells were fixed, permeabilized and labeled with specific primary antibodies before using Alexa Fluor 488-conjugated (green) and Alexa Fluor 647-conjugated (purple) secondary antibodies. Z-stack of fluorescence images were acquired for RNF13-HA (in purple) with endogenous markers (green). (**S1A)** The results demonstrate limited colocalization between Protein disulfide-isomerase (PDI), a marker for endogenous ER-

localized proteins, and RNF13 suggesting that HA-tagged WT RNF13 and its L311S and L312P variant proteins do not extensively localize to the ER. (**S1B**) To eliminate the possibility that RNF13 variants L311S and L312P relocate to mitochondria, HeLa cells expressing HA-tagged RNF13 proteins were co-labeled with the endogenous mitochondrial marker Apoptosis-inducing factor (AIF). The results show that RNF13 proteins, WT and its variants L311S and L312P, do not colocalize with AIF. (**S1C**) RNF13 is a known highly glycosylated type I integral membrane protein that goes through the Golgi apparatus after its synthesis in the ER [1,2]. Thus, to ensure that the trafficking of RNF13 through the Golgi apparatus is not affected by the presence of L311S and L312P variants, HeLa cells were co-labeled for HA-tagged RNF13 and the Receptor binding cancer antigen expressed in SiSo cells (RCAS1) transmembrane Golgi protein. The results show that fluorescence from neither RNF13 WT nor the variants displayed the characteristic structure of the Golgi apparatus and did not colocalize extensively with RCAS1, indicating that these proteins do not remain in the Golgi. Scale bars indicate 10 µm for whole cell image and 2 µm for boxed inset.

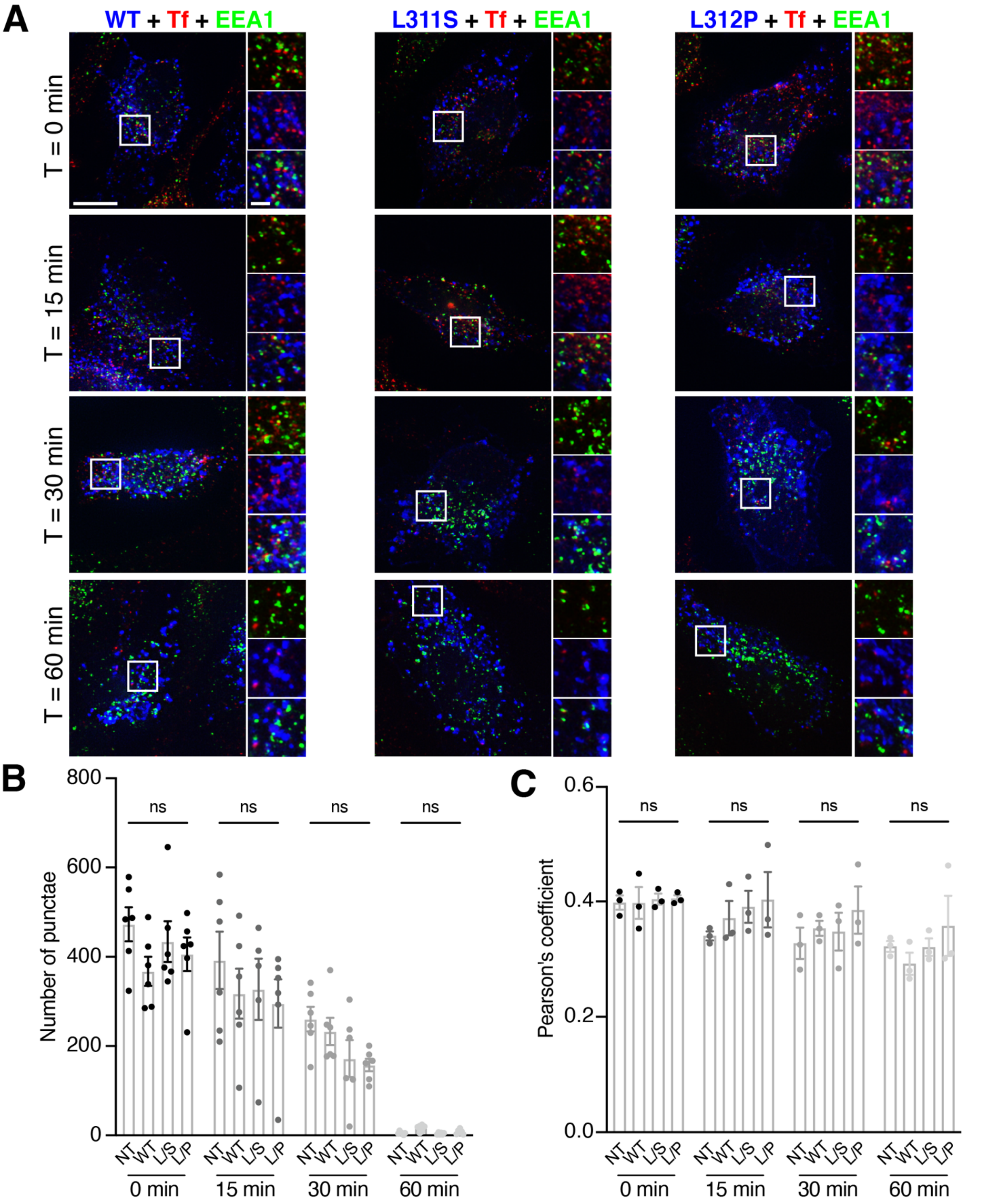

Figure S2. RNF13 variants L311S and L312P alter transferrin recycling. Starved HeLa cells transiently expressing HA-tagged RNF13 WT, L311S or L312P were incubated with

transferrin for 5 minutes. Cells were washed and incubated in fresh culture medium for the indicated time before being fixed, permeabilized and labeled with specific primary antibody against HA (RNF13) and endogenous EEA1. (**S2A**) Representative images are show transferrin (Tf, red) with RNF13 (blue) and early endosome marker EEA1 (green). Scale bar = 10 μm. Boxed areas are shown at higher magnification (scale bar = 2 μm). (**S2B**) Quantitation of the average number of Tf puncta per cell. The bar graph presents the mean ± SEM while the scatter plot represents the distribution of the average number of Tf puncta for each independent experiment. Analyses were performed on 6-10 cells within 5 fields-of-view collected for each of the six independent experiments (N=6, total of 31 to 45 cells per condition). Not significant (ns) using two-way ANOVA with multiple comparisons. (**S2C**) Quantitation of overlap between Tf and EEA1. The bar graph presents the mean ± SEM while the scatter plot represents the distribution of the averaged Pearson's coefficient of each independent experiment analyzed (N=3). Each condition contains 35 to 42 cells. Not significant (ns) using two-way ANOVA with multiple comparisons.

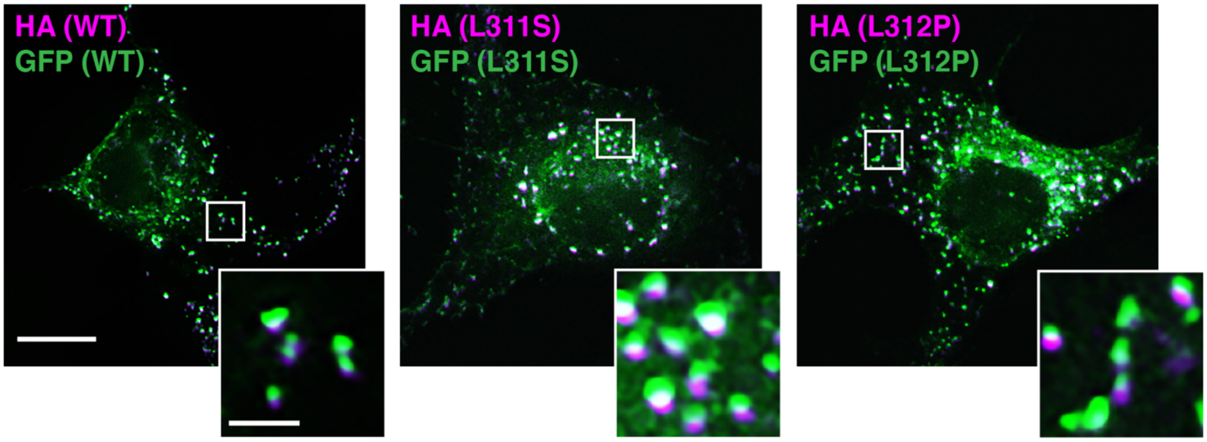

Figure S3. Localization of RNF13 is not affected by the presence of either HA- or GFP-tag. Previous studies showed an extensive ER localization for RNF13 [1,3] thus we tested the influence of the HA-tag under our conditions. HeLa cells were co-transfected with both HA-tagged RNF13 and GFP-tagged RNF13, a construct used by other group [4]. Cells were fixed, permeabilized and labeled with specific primary antibodies against HA or GFP. Representative images from two independent experiments (N=2) show RNF13-HA (in purple) and RNF13-GFP (in green). Scale bars indicate 10 µm for whole cell image and 2 µm for boxed inset. Our results show an extensive colocalization between both tagged proteins without any obvious difference in the overall intracellular localization of RNF13.

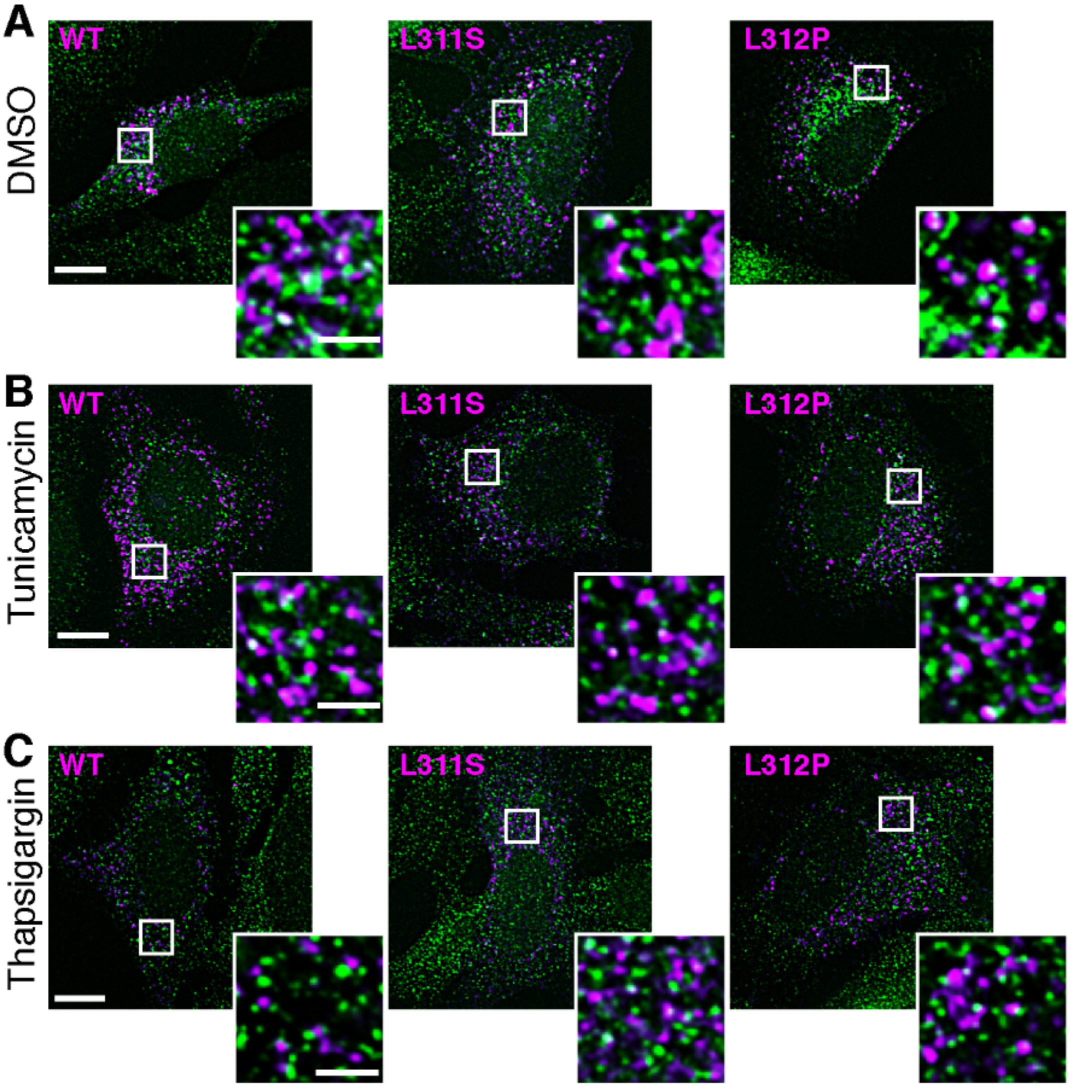

Figure S4. Chemically induced endoplasmic reticulum stress does not promote extensive ER localization of RNF13 in HeLa cells. RNF13 is known to activate the Jun NH2-terminal Kinase (JNK) pathway and induce apoptosis during ER stress [3]. Tunicamycin (Tn)-induced ER stress in cells expressing the RNF13 L311S mutant also increases the level of apoptosis markers, such as phosphorylated c-Jun and spliced X-box binding protein 1 (XPB1), resulting in the hypothesis that the genetic variant proteins confer a gain-of-function and alter apoptosis signalling from the ER stress response [5]. Thus far, our data show that neither RNF13 WT nor the mutant proteins extensively localize to the ER (Fig. S1A). Nonetheless, we tested the possibility that RNF13 variants proteins could be relocating to or be trapped in the

ER following drug-induced ER stress [3,5]. (**Fig. S4A-C**) Transfected HeLa cells were treated for 3 hours with DMSO (**S4A**), 1.25 µg/ml tunicamycin (Tn) (**S4B**) or 1 µM thapsigargin (Tg) (**S4C**). Cells were fixed, permeabilized and then labeled with specific primary antibody against HA (RNF13, in purple) and for endogenous ER labeling (PDI, in green). Z-stack of fluorescence images were acquired by fluorescence microscopy. Representative images were selected from three independent experiments (N=3), and the boxed area is shown at higher magnification. Scale bars indicate 10 µm for whole cell image and 2 µm for the boxed inset. The results demonstrate that Tn and Tg applications do not increase colocalization between PDI and RNF13 when compared to DMSO. Under the tested conditions, our results demonstrate that RNF13 WT and mutants L311S and L312P do not localize in the ER under basal or drug-induced stress conditions.

SUPPLEMENTARY FIGURES REFERENCES

1. Zhang, Q.; Meng, Y.; Zhang, L.; Chen, J.; Zhu, D. RNF13: a novel RING-type ubiquitin ligase over-expressed in pancreatic cancer. *Cell Res* **2009**, *19*, 348-357, doi:10.1038/cr.2008.285.
2. Bocock, J.P.; Carmicle, S.; Chhotani, S.; Ruffolo, M.R.; Chu, H.; Erickson, A.H. The PA-TM-RING protein RING finger protein 13 is an endosomal integral membrane E3 ubiquitin ligase whose RING finger domain is released to the cytoplasm by proteolysis. *The FEBS journal* **2009**, *276*, 1860-1877, doi:10.1111/j.1742-4658.2009.06913.x.
3. Arshad, M.; Ye, Z.; Gu, X.; Wong, C.K.; Liu, Y.; Li, D.; Zhou, L.; Zhang, Y.; Bay, W.P.; Yu, V.C., et al. RNF13, a RING finger protein, mediates endoplasmic reticulum stress-induced apoptosis through the inositol-requiring enzyme (IRE1α)/c-Jun NH2-terminal kinase pathway. *J Biol Chem* **2013**, *288*, 8726-8736, doi:10.1074/jbc.M112.368829.
4. van Dijk, J.R.; Yamazaki, Y.; Palmer, R.H. Tumour-associated mutations of PA-TM-RING ubiquitin ligases RNF167/RNF13 identify the PA domain as a determinant for endosomal localization. *Biochem J* **2014**, *459*, 27-36, doi:10.1042/bj20131067.
5. Edvardson, S.; Nicolae, C.M.; Noh, G.J.; Burton, J.E.; Punzi, G.; Shaag, A.; Bischetsrieder, J.; De Grassi, A.; Pierri, C.L.; Elpeleg, O., et al. Heterozygous RNF13 Gain-of-Function Variants Are Associated with Congenital Microcephaly, Epileptic Encephalopathy, Blindness, and Failure to Thrive. *Am J Hum Genet* **2019**, *104*, 179-185, doi:10.1016/j.ajhg.2018.11.018.
