# Supplementary material for: RNF13 Dileucine Motif Variants L311S and L312P Interfere with Endosomal Localization and AP-3 Complex Association
Source: Cells. 2021 Nov 6;10(11):3063. doi: 10.3390/cells10113063 (PMC8620429; doi:10.3390/cells10113063)
Supplement: Supplementary file 1 [file cells-10-03063-s001.zip › Cabana et al 2021_ supplementary tables S1-S10 final.pdf]

SUPPLEMENTARY TABLES S1-10

RNF13 dileucine motif variants L311S and L312P interfere with endosomal localization and AP-3 complex association

Valérie C. Cabana, Antoine Y. Bouchard, Audrey M. Sénécal, Kim Ghilarducci, Saïd Kourrich, Laurent Cappadocia, Marc P. Lussier

Table S1. Statistical values - Manders overlap coefficient, Figure 1A-E

Marker		RNF13 WT	RNF13 L311S	RNF13 L312P
**EEA1** (panel A)	Number of cells analyzed (after removal of outliers)	29	30	30
	Mean ± SEM	0.0825 ± 0.0075	0.0849 ± 0.0076	0.0973 ± 0.0070
	Exact p value (WT)		>0.9999	0.3167
	Exact p value (L311S)	>0.9999		0.6795
	Exact p value (L312P)	0.3167	0.6795	
**Lamp 1** (panel B)	Number of cells analyzed (after removal of outliers)	29	30	30
	Mean ± SEM	0.2778 ± 0.0152	0.1435 ± 0.0151	0.1726 ± 0.0160
	Exact p value (WT)		<0.0001	0.0004
	Exact p value (L311S)	<0.0001		0.6402
	Exact p value (L312P)	0.0004	0.6402	
**Rab5** (panel C)	Number of cells analyzed (after removal of outliers)	30	29	30
	Mean ± SEM	0.2141 ± 0.0186	0.2210 ± 0.0198	0.2068 ± 0.0169
	Exact p value (WT)		>0.9999	>0.9999
	Exact p value (L311S)	>0.9999		>0.9999
	Exact p value (L312P)	>0.9999	>0.9999	
**Rab7** (panel D)	Number of cells analyzed (after removal of outliers)	30	30	30
	Mean ± SEM	0.3669 ± 0.0188	0.3224 ± 0.0228	0.3145 ± 0.0195
	Exact p value (WT)		0.2209	0.2197
	Exact p value (L311S)	0.2209		>0.9999
	Exact p value (L312P)	0.2197	>0.9999	
**Rab9** (panel E)	Number of cells analyzed (after removal of outliers)	30	30	30
	Mean ± SEM	0.3606 ± 0.0275	0.3419 ± 0.0196	0.3731 ± 0.0205
	Exact p value (WT)		>0.9999	>0.9999
	Exact p value (L311S)	>0.9999		0.9260
	Exact p value (L312P)	>0.9999	0.9260	

Table S2. Statistical values - Vesicle diameter, Figure 1F-J

Marker		NT	RNF13 WT	RNF13 L311S	RNF13 L312P
**EEA1** (panel F)	Number of vesicles analyzed (after removal of outliers)	75	71	75	74
	Mean ± SEM (µm)	0.4048 ± 0.0089	0.3877 ± 0.0093	0.5119 ± 0.0126	0.5151 ± 0.0100
	Exact p value (NT)		>0.9999	<0.0001	<0.0001
	Exact p value (WT)	>0.9999		<0.0001	<0.0001
	Exact p value (L311S)	<0.0001	<0.0001		>0.9999
	Exact p value (L312P)	<0.0001	<0.0001	>0.9999	
**Lamp 1** (panel G)	Number of vesicles analyzed (after removal of outliers)	75	75	74	73
	Mean ± SEM (µm)	0.6415 ± 0.0124	0.6365 ± 0.0138	0.8104 ± 0.0238	0.7541 ± 0.0183
	Exact p value (NT)		>0.9999	<0.0001	<0.0001
	Exact p value (WT)	>0.9999		<0.0001	<0.0001
	Exact p value (L311S)	<0.0001	<0.0001		>0.9999
	Exact p value (L312P)	<0.0001	<0.0001	>0.9999	
**Rab5** (panel H)	Number of vesicles analyzed (after removal of outliers)	75	75	75	75
	Mean ± SEM (µm)	0.5175 ± 0.0106	0.5161 ± 0.0083	0.6325 ± 0.0088	0.6520 ± 0.0089
	Exact p value (NT)		>0.9999	<0.0001	<0.0001
	Exact p value (WT)	>0.9999		<0.0001	<0.0001
	Exact p value (L311S)	<0.0001	<0.0001		>0.9999
	Exact p value (L312P)	<0.0001	<0.0001	>0.9999	
**Rab7** (panel I)	Number of vesicles analyzed (after removal of outliers)	75	75	75	75
	Mean ± SEM (µm)	0.7512 ± 0.0128	0.7679 ± 0.0122	1.061 ± 0.0170	1.012 ± 0.0144
	Exact p value (NT)		>0.9999	<0.0001	<0.0001
	Exact p value (WT)	>0.9999		<0.0001	<0.0001
	Exact p value (L311S)	<0.0001	<0.0001		>0.9999
	Exact p value (L312P)	<0.0001	<0.0001	>0.9999	
**Rab9** (panel J)	Number of vesicles analyzed (after removal of outliers)	75	75	75	75
	Mean ± SEM (µm)	0.7947 ± 0.0115	0.8017 ± 0.0108	0.6149 ± 0.0090	0.6089 ± 0.0101
	Exact p value (NT)		>0.9999	<0.0001	<0.0001
	Exact p value (WT)	>0.9999		<0.0001	<0.0001
	Exact p value (L311S)	<0.0001	<0.0001		>0.9999
	Exact p value (L312P)	<0.0001	<0.0001	>0.9999	

Table S3. Statistical values - Number of punctae (EGF), Figure 2B

Time		NT	RNF13 WT	RNF13 L311S	RNF13 L312P
0 min	Number of cells analyzed (after removal of outliers)	44	41	45	43
	Mean ± SEM	264 ± 19	221 ± 20	220 ± 41	228 ± 38
	Exact p value (NT)		0.3787	0.3478	0.9996
	Exact p value (WT)	0.3787		>0.9999	0.4402
	Exact p value (L311S)	0.3478	>0.9999		0.4071
	Exact p value (L312P)	0.9996	0.4402	0.4071	
15 mins	Number of cells analyzed (after removal of outliers)	44	46	48	43
	Mean ± SEM	157 ± 14	154 ± 10	152 ± 14	156 ± 16
	Exact p value (NT)		0.9994	0.9978	>0.9999
	Exact p value (WT)	0.9994		>0.9999	0.9998
	Exact p value (L311S)	0.9978	>0.9999		0.9988
	Exact p value (L312P)	>0.9999	0.9998	0.9988	
30 mins	Number of cells analyzed (after removal of outliers)	46	46	45	42
	Mean ± SEM	108 ± 7	115 ± 9	98 ± 7	103 ± 8
	Exact p value (NT)		0.9939	0.9788	0.9976
	Exact p value (WT)	0.9939		0.9138	0.9701
	Exact p value (L311S)	0.9788	0.9138		0.9968
	Exact p value (L312P)	0.9976	0.9701	0.9968	
60 mins	Number of cells analyzed (after removal of outliers)	46	47	50	45
	Mean ± SEM	55 ± 5	76 ± 9	84 ± 7	83 ± 6
	Exact p value (NT)		0.8489	0.6785	0.7015
	Exact p value (WT)	0.8489		0.9898	0.9930
	Exact p value (L311S)	0.6785	0.9898		>0.9999
	Exact p value (L312P)	0.7015	0.9930	>0.9999	

Table S4. Statistical values - Pearson's coefficient (EGF), Figure 2C

Time		NT	RNF13 WT	RNF13 L311S	RNF13 L312P
0 min	Number of cells analyzed (after removal of outliers)	45	40	38	42
	Mean ± SEM	0.363 ± 0.008	0.355 ± 0.012	0.307 ± 0.005	0.282 ± 0.010
	Exact p value (NT)		0.9558	0.0041	<0.0001
	Exact p value (WT)	0.9558		0.0155	0.0002
	Exact p value (L311S)	0.0041	0.0155		0.3568
	Exact p value (L312P)	<0.0001	0.0002	0.3568	
15 mins	Number of cells analyzed (after removal of outliers)	45	45	44	42
	Mean ± SEM	0.222 ± 0.005	0.259 ± 0.013	0.275 ± 0.003	0.275 ± 0.022
	Exact p value (NT)		0.0927	0.0070	0.0066
	Exact p value (WT)	0.0927		0.6983	0.6850
	Exact p value (L311S)	0.0070	0.6983		>0.9999
	Exact p value (L312P)	0.0066	0.6850	>0.9999	
30 mins	Number of cells analyzed (after removal of outliers)	45	37	44	42
	Mean ± SEM	0.155 ± 0.014	0.239 ± 0.030	0.214 ± 0.020	0.202 ± 0.006
	Exact p value (NT)		0.0093	0.0020	0.0173
	Exact p value (WT)	0.0093		0.9379	0.9948
	Exact p value (L311S)	0.0020	0.9379		0.8432
	Exact p value (L312P)	0.0173	0.9948	0.8432	
60 mins	Number of cells analyzed (after removal of outliers)	45	45	41	43
	Mean ± SEM	0.073 ± 0.004	0.077 ± 0.005	0.093 ± 0.008	0.082 ± 0.004
	Exact p value (NT)		0.9932	0.5486	0.9312
	Exact p value (WT)	0.9932		0.7115	0.9870
	Exact p value (L311S)	0.5486	0.7115		0.8827
	Exact p value (L312P)	0.9312	0.9870	0.8827	

Table S5. Statistical values - Number of punctae (Tf) Figure S2B

Time		NT	RNF13 WT	RNF13 L311S	RNF13 L312P
0 min	Number of cells analyzed (after removal of outliers)	40	43	39	44
	Mean ± SEM	473 ± 38	368 ± 33	434 ± 46	406 ± 38
	Exact p value (NT)		0.2133	0.8888	0.6000
	Exact p value (WT)	0.2133		0.6058	0.8925
	Exact p value (L311S)	0.8888	0.6058		0.9527
	Exact p value (L312P)	0.6000	0.8925	0.9527	
15 mins	Number of cells analyzed (after removal of outliers)	42	44	36	45
	Mean ± SEM	392 ± 64	317 ± 56	327 ± 69	295 ± 54
	Exact p value (NT)		0.5126	0.6643	0.2830
	Exact p value (WT)	0.5126		0.9980	0.9764
	Exact p value (L311S)	0.6643	0.9980		0.9409
	Exact p value (L312P)	0.2830	0.9764	0.9409	
30 mins	Number of cells analyzed (after removal of outliers)	40	42	42	44
	Mean ± SEM	261 ± 28	233 ± 30	172 ± 41	158 ± 14
	Exact p value (NT)		0.9557	0.3634	0.2347
	Exact p value (WT)	0.9557		0.6752	0.5088
	Exact p value (L311S)	0.3634	0.6752		0.9934
	Exact p value (L312P)	0.2347	0.5088	0.9934	
60 mins	Number of cells analyzed (after removal of outliers)	31	36	34	33
	Mean ± SEM	5 ± 1	16 ± 3	4 ± 1	8 ± 2
	Exact p value (NT)		0.9968	>0.9999	>0.9999
	Exact p value (WT)	0.9968		0.9957	0.9985
	Exact p value (L311S)	>0.9999	0.9957		0.9999
	Exact p value (L312P)	>0.9999	0.9985	0.9999	

Table S6. Statistical values - Pearson's coefficient (Tf), Figure S2C

Time		NT	RNF13 WT	RNF13 L311S	RNF13 L312P
0 min	Number of cells analyzed (after removal of outliers)	42	38	39	42
	Mean ± SEM	0.398 ± 0.012	0.397 ± 0.028	0.404 ± 0.009	0.405 ± 0.006
	Exact p value (NT)		>0.9999	0.9987	0.9979
	Exact p value (WT)	>0.9999		0.9982	0.9972
	Exact p value (L311S)	0.9987	0.9982		>0.9999
	Exact p value (L312P)	0.9979	0.9972	>0.9999	
15 mins	Number of cells analyzed (after removal of outliers)	40	41	36	38
	Mean ± SEM	0.340 ± 0.008	0.371 ± 0.030	0.391 ± 0.028	0.403 ± 0.048
	Exact p value (NT)		0.8595	0.5741	0.3878
	Exact p value (WT)	0.8595		0.9573	0.8437
	Exact p value (L311S)	0.5741	0.9573		0.9888
	Exact p value (L312P)	0.3878	0.8437	0.9888	
30 mins	Number of cells analyzed (after removal of outliers)	43	39	41	40
	Mean ± SEM	0.328 ± 0.027	0.353 ± 0.013	0.348 ± 0.033	0.385 ± 0.041
	Exact p value (NT)		0.9115	0.9552	0.4654
	Exact p value (WT)	0.9115		0.9989	0.8477
	Exact p value (L311S)	0.9552	0.9989		0.7734
	Exact p value (L312P)	0.4654	0.8477	0.7734	
60 mins	Number of cells analyzed (after removal of outliers)	41	40	35	42
	Mean ± SEM	0.322 ± 0.009	0.292 ± 0.019	0.321 ± 0.015	0.358 ± 0.052
	Exact p value (NT)		0.8671	>0.9999	0.7965
	Exact p value (WT)	0.8671		0.8818	0.3472
	Exact p value (L311S)	>0.9999	0.8818		0.7781
	Exact p value (L312P)	0.7965	0.3472	0.7781	

Table S7. Statistical values – Manders overlap coefficient, Figure 3A-C

Marker		RNF13 WT	RNF13 LL / AA
**EEA1** (panel A)	Number of cells analyzed (after removal of outliers)	30	28
	Mean ± SEM	0.07313 ± 0.00614	0.05979 ± 0.00673
	Exact p value		0.0631
**Lamp 1** (panel B)	Number of cells analyzed (after removal of outliers)	30	29
	Mean ± SEM	0.3095 ± 0.0209	0.1688 ± 0.0195
	Exact p value		<0.0001
**Rab7** (panel C)	Number of cells analyzed (after removal of outliers)	30	30
	Mean ± SEM	0.2866 ± 0.0154	0.2794 ± 0.0154
	Exact p value		0.5945

Table S8. Statistical values – Vesicle diameter, Figure 3D-F

**Marker**		**NT**	**RNF13 WT**	**RNF13 LL / AA**
**EEA1** (panel D)	Number of vesicles analyzed (after removal of outliers)	75	75	75
	Mean ± SEM (µm)	0.4747 ± 0.0079	0.4701 ± 0.0082	0.5828 ± 0.0112
	Exact p value (NT)		>0.9999	<0.0001
	Exact p value (WT)	>0.9999		<0.0001
	Exact p value (LL/AA)	<0.0001	<0.0001	
**Lamp 1** (panel E)	Number of vesicles analyzed (after removal of outliers)	73	75	75
	Mean ± SEM (µm)	0.6129 ± 0.0110	0.6307 ± 0.0118	0.7957 ± 0.0131
	Exact p value (NT)		0.9636	<0.0001
	Exact p value (WT)	0.9636		<0.0001
	Exact p value (LL/AA)	<0.0001	<0.0001	
**Rab7** (panel F)	Number of vesicles analyzed (after removal of outliers)	75	75	75
	Mean ± SEM (µm)	0.6941 ± 0.0108	0.7197 ± 0.0132	0.8659 ± 0.0118
	Exact p value (NT)		0.5839	<0.0001
	Exact p value (WT)	0.5839		<0.0001
	Exact p value (LL/AA)	<0.0001	<0.0001	

Table S9. Statistical values – Manders overlap coefficient, Figure 6C and 6F

Marker		CTL	si#1	si#2
**Lamp1** (panel C)	Number of cells analyzed (after removal of outliers)	30	30	30
	Mean ± SEM	0.3161 ± 0.0207	0.1869 ± 0.0195	0.1635 ± 0.0165
	Exact p value (CTL)		0.0001	<0.0001
	Exact p value (si#1)	0.0001		>0.9999
	Exact p value (si#2)	<0.0001	>0.9999	
**Rab7** (panel F)	Number of cells analyzed (after removal of outliers)	29	30	30
	Mean ± SEM	0.3722 ± 0.0324	0.3221 ± 0.0265	0.3389 ± 0.0277
	Exact p value (CTL)		>0.9999	>0.9999
	Exact p value (si#1)	>0.9999		>0.9999
	Exact p value (si#2)	>0.9999	>0.9999	

Table S10. Statistical values – Vesicle diameter, Figure 6D and G

Marker		CTL+ vector	CTL+ WT	si#1+ vector	si#1+ WT	si#2+ vector	si#2+ WT
**Lamp1** (panel D)	Number of vesicles analyzed (after removal of outliers)	75	72	75	75	75	73
	Mean ± SEM (µm)	0.6621 ± 0.0118	0.6244 ± 0.0107	0.8384 ± 0.0150	0.8488 ± 0.0151	0.8296 ± 0.0126	0.8338 ± 0.0136
	Exact p value (CTL+vector)		>0.9999	<0.0001	<0.0001	<0.0001	<0.0001
	Exact p value (CTL+WT)	>0.9999		<0.0001	<0.0001	<0.0001	<0.0001
	Exact p value (si#1+vector)	<0.0001	<0.0001		>0.9999	>0.9999	>0.9999
	Exact p value (si#1+WT)	<0.0001	<0.0001	>0.9999		>0.9999	>0.9999
	Exact p value (si#2+vector)	<0.0001	<0.0001	>0.9999	>0.9999		>0.9999
	Exact p value (si#2+WT)	<0.0001	<0.0001	>0.9999	>0.9999	>0.9999	
**Rab7** (panel G)	Number of vesicles analyzed (after removal of outliers)	75	75	75	75	75	75
	Mean ± SEM (µm)	0.7037 ± 0.0116	0.7183 ± 0.0102	0.8689 ± 0.0115	0.8692 ± 0.0133	0.8577 ± 0.0116	0.8637 ± 0.0133
	Exact p value (CTL+vector)		>0.9999	<0.0001	<0.0001	<0.0001	<0.0001
	Exact p value (CTL+WT)	>0.9999		<0.0001	<0.0001	<0.0001	<0.0001
	Exact p value (si#1+vector)	<0.0001	<0.0001		>0.9999	>0.9999	>0.9999
	Exact p value (si#1+WT)	<0.0001	<0.0001	>0.9999		>0.9999	>0.9999
	Exact p value (si#2+vector)	<0.0001	<0.0001	>0.9999	>0.9999		>0.9999
	Exact p value (si#2+WT)	<0.0001	<0.0001	>0.9999	>0.9999	>0.9999	
